# A multimodal EMG–IMU dataset and multi-dataset benchmark for deep learning-based human activity recognition

**DOI:** 10.1038/s41598-026-64847-4

**Published:** 2026-08-20

**Authors:** Mohamed Khaled Farouk, Mohamed Fawzy El-Khatib, Mohammed I. Awad, Mostafa R. A. Atia, A. Abdellatif

**Affiliations:** 1 Mechatronics and Robotics Engineering Department, Faculty of Engineering, Egyptian Russian University, Cairo, 11829 Egypt; 2 Mechatronics Engineering Department, Faculty of Engineering, Ain Shams University, Cairo, 11517 Egypt; 3 Mechanical Engineering Department, Arab Academy for Science Technology and Maritime Transport, Sheraton Branch, Cairo, 11757 Egypt

**Keywords:** Human activity recognition, Wearable sensors, Electromyography and inertial measurement fusion, Deep learning, Hybrid convolutional neural network–long short-term memory models, Generative adversarial network, Computational biology and bioinformatics, Engineering, Mathematics and computing

## Abstract

Human Activity Recognition (HAR) using wearable sensors is relevant to rehabilitation, assistive robotics, and mobile health applications. This study presents (i) SDALLE, a publicly available multimodal dataset integrating surface electromyography (EMG) and inertial measurement unit (IMU) signals acquired using a DELSYS Trigno wireless system; (ii) a harmonized, subject-dependent comparison with the public ENABL3S dataset; and (iii) an exploratory Wasserstein Generative Adversarial Network with Gradient Penalty (WGAN-GP) augmentation strategy for class balancing. SDALLE contains recordings of nine healthy male subjects walking, jogging, climbing stairs and descending stairs. EMG and IMU signals were synchronized, standardized, and segmented using 75%-overlapping windows. In the implemented pipeline, feature standardization preceded segmentation, after which the windows were randomly divided into 70% training and 30% testing subsets without participant- or trial-level grouping. Consequently, overlapping windows, including windows from the same participant and trial, could occur in both subsets. Under this window-level protocol, attention-enhanced CNN–LSTM models recorded approximately 98–99% accuracy on ENABL3S and approximately 99% on SDALLE. These values are protocol-specific and may be optimistic; they do not measure generalization to unseen participants or establish a portable model ranking. GAN augmentation increased the SDALLE row count by approximately 12% and changed average testing accuracy from 96.9% before augmentation to 96.5% afterward under the implemented protocol. The study therefore provides a dataset resource and an exploratory subject-dependent comparison of predictive and computational behavior. Participant-independent evaluation with training-fold-only normalization, model selection, and GAN training is required before drawing conclusions about cross-subject robustness or deployment readiness.

**Supplementary Information:**

The online version contains supplementary material available at 10.1038/s41598-026-64847-4.

## Introduction

Human Activity Recognition (HAR) based on wearable sensing technologies has become a fundamental element in rehabilitation engineering^1^, assistive robotics^2^ and prosthetic systems^3,4^, human–robot interaction^5^, and mobile health monitoring^6^. Accordingly, this study focuses specifically on lower-limb locomotor activities that are highly relevant to rehabilitation, prosthetic control, gait assessment, and wearable assistive systems. Reliable recognition of locomotion activities allows for continuous assessment of functional mobility, early detection of pathological gait patterns^7^ and adaptive control of assistive devices running in real-world environments. Compared with vision-based approaches, wearable sensor–based HAR presents some practical advantages for deployment, such as improved privacy preservation, robustness to occlusion and illumination variability, and feasibility for real-time on-body inference^8,9^. Of the sensing modalities, the combination of surface electromyography (EMG) and inertial measurement units (IMUs) is of special interest. EMG measures the neuromuscular activation while IMUs measure the resultant kinematics of the body segments^10,11^. Because there is an electromechanical delay between muscle activation and visible motion, EMG offers an earlier indication of movement intention, which is very important for responsive rehabilitation and assistive control systems^12^.

With the advent of deep learning, HAR research has moved from handcrafted feature engineering^13^ to end-to-end representation learning from raw or minimally processed time-series signals^14,15^. Convolutional Neural Networks (CNNs) have shown to be powerful in extracting discriminative local features from multichannel sensor windows, both in one-dimensional temporal and two-dimensional pseudo-image representations^16^. Long Short-Term Memory (LSTM) networks are able to model longer temporal dependencies in sequential data^17^. Hybrid CNN-LSTM architectures combine spatial feature extraction with temporal modelling^18^ and attention mechanisms further improve recognition by highlighting informative temporal segments^19,20^.

In our previous study using the publicly available ENABL3S dataset^20,28^, we conducted a systematic benchmarking of Artificial Neural Networks (ANN), CNN, LSTM, CNN–LSTM, and attention-enhanced models. The attention-based CNN-LSTM architectures increased the classification accuracy, but also increased the computational complexity. This observation raised an important deployment-oriented question: when does the additional architectural complexity justify its computational overhead in wearable and embedded systems constrained by memory, latency, and processing power.

Despite progress in the methodology, two critical limitations still constrain the development of robust and deployable multimodal HAR systems. First, many studies evaluate the models on one dataset, limiting the insight on cross dataset benchmarking. Inter-subject variability, differences in sensor placement and acquisition noise can significantly affect the signal characteristics in wearable sensing. Generalisability is a crucial problem. Second, the trade-off between recognition accuracy and computational efficiency is often underexplored in a way that is directly relevant to real-time, on-device inference.

Public benchmark datasets such as KU-HAR^21,22^, UCI HAR^23,24^, WISDM^25,26^, PAMAP2^27^ and ENABL3S^28,29^ have enabled reproducible evaluation in wearable HAR. However, many rely primarily on inertial sensing, provide limited physiological information, or lack detailed documentation regarding synchronization and preprocessing. Furthermore, a number of popular datasets have poor coverage of elevation transition locomotor activities such as stair ascent and descent which are of particular interest for rehabilitation and assistive applications. In recent years, several multimodal locomotion datasets combining EMG and inertial sensing have been proposed for wearable Human Activity Recognition (HAR) and gait analysis research^30–33^. These datasets differ significantly with respect to participant population, sensing configuration, locomotor activity coverage, sampling frequency and benchmarking objectives. Some datasets are dedicated to high-density biomechanical acquisition and clinical gait analysis^31^, others mainly to large scale inertial sensing or activity monitoring^32,33^. Table 1: Summary of representative datasets for multimodal locomotion and relative position of the proposed SDALLE dataset. In contrast with many existing resources, SDALLE integrates synchronised multimodal EMG–IMU acquisition with a unified preprocessing and deep learning benchmarking framework for reproducible comparative evaluation of wearable locomotor HAR architectures.

**Table 1:** Comparison of SDALLE with representative multimodal locomotion datasets.

Feature	ENABL3S	Dimitrov et al.	Gait120	SDALLE (Proposed)
Participants	10	Multiple	120	9
EMG Configuration	14 channels	HD-EMG (64 ch)	Limited	Standard EMG
IMU Sensors	Yes	Yes	Yes	Yes
Activities	6 + transitions	8 + activities	Gait activities	4 locomotor tasks
Sampling Rate	1,000 Hz (EMG) 500 Hz (IMU and kinematic signals)	2000 Hz	Variable	1259/148 Hz
Public Availability	Yes	Yes	Yes	Yes
Deep Learning Benchmark	Partial	No	Limited	Unified benchmark
Primary Focus	Locomotion analysis	High-density acquisition	Large-scale gait	Multimodal HAR benchmarking

SDALLE complements a number of existing datasets that focus mainly on biomechanical acquisition by offering a harmonised benchmarking framework for controlled comparison of different deep learning architectures with uniform preprocessing and evaluation. Additional challenges include data scarcity and class imbalance, especially in a multimodal setting with high-quality EMG acquisition. Limited sample sizes and skewed activity distributions may induce bias during model training and reduce robustness. While conventional augmentation techniques (e.g. noise injection, scaling, window perturbation) increase variability, they do not generate fundamentally new signal realisations. Generative Adversarial Networks (GANs) have recently emerged as a data-driven alternative for synthesising realistic time-series signals and have shown promise in physiological sensing applications^30^. Their practical benefits for multimodal EMG–IMU HAR, however, are not yet clearly demonstrated, especially if the performance gains are due to the improvement of accuracy or due to the improvement of class balance and bias mitigation.

Motivated by these gaps, this study proposes SDALLE, a novel publicly available EMG–IMU locomotion dataset recorded using a wireless DELSYS Trigno system, and presents a harmonised subject-dependent comparison with ENABL3S. Because preprocessing and randomised partitioning were done at the window level rather than the participant level, the resulting comparison is interpreted as protocol-specific and exploratory rather than as evidence of generalisation to unseen participants.

The contribution of this work is not to propose completely new deep learning architectures. Instead, the novelty primarily resides in:

1. SDALLE multimodal EMG-IMU locomotion dataset, with synchronised acquisition and well-documented preprocessing pipelines, available on public domain.
2. Development of a common comparison of established deep learning architectures, under the same conditions of preprocessing, segmentation, training and window-level evaluation, noting that the protocol does not provide participant-independent validation.
3. A descriptive analysis of the trade-off between measured recognition performance and computational complexity for different families of architectures. The analysis is not a validation of the deployment readiness.
4. Structured exploratory investigation of GAN-based augmentation: Evaluation of class-distribution and downstream classifier behaviour under the same subject-dependent window-level protocol, without inferring cross-subject robustness or prediction stability.

## Methodology

**Fig. 1:**
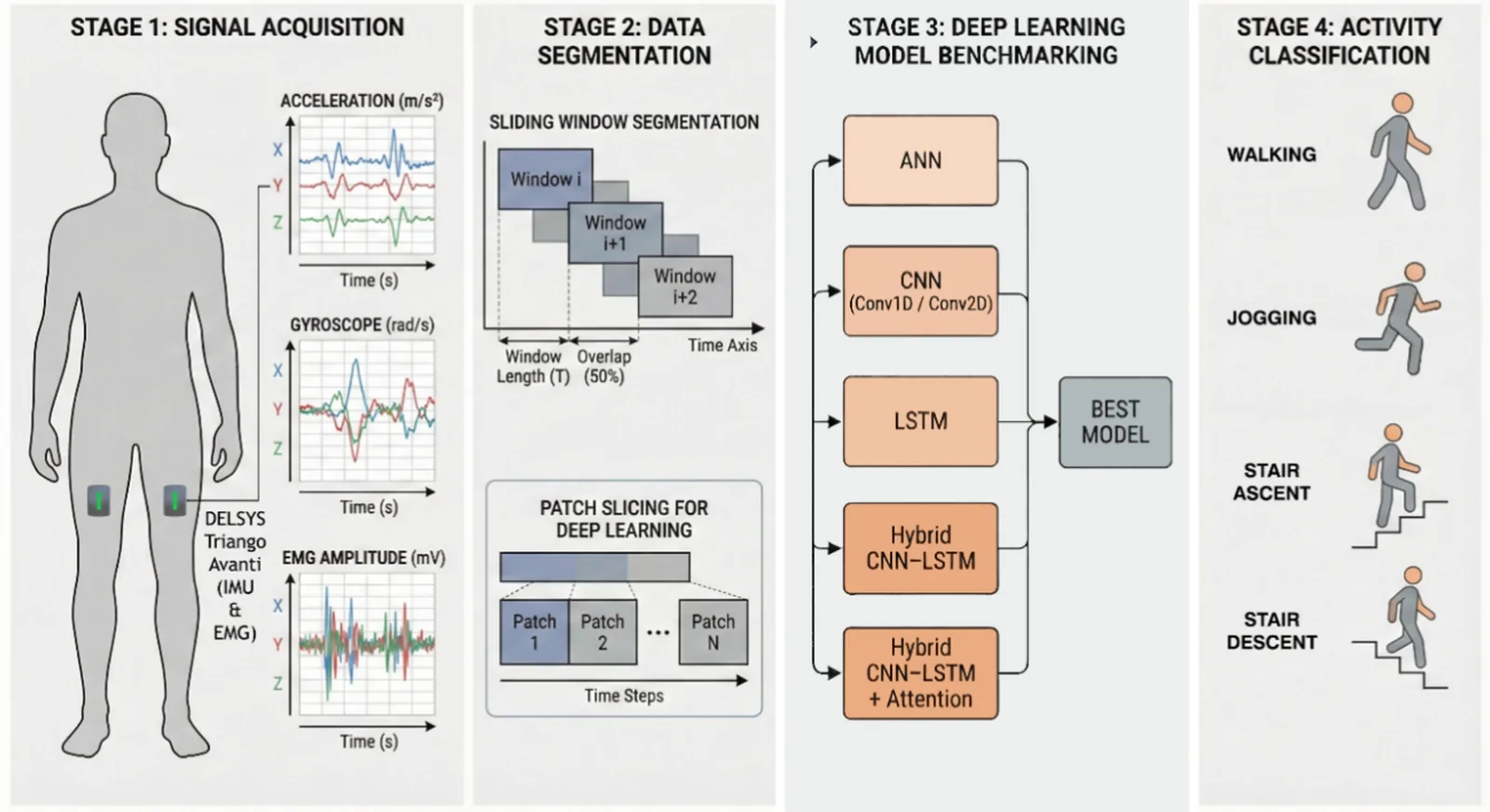
Overview of the proposed electromyography–inertial measurement–based Human Activity Recognition pipeline.

This section describes the experimental setting of the Human Activity Recognition based on multimodal electromyography-inertial measurement. The pipeline consists of signal acquisition, preprocessing and segmentation, benchmarking of deep learning models and multi-class activity classification. During walking, jogging, stair ascent and stair descent tasks, simultaneous surface electromyography and inertial measurement signals were recorded. The recorded multichannel data were aligned, standardised and segmented into fixed length samples with overlapping sliding windows. Consistent optimisation settings were used to train multiple architectures, including Artificial Neural Networks, one- and two-dimensional Convolutional Neural Networks, Long Short-Term Memory networks, hybrid CNN–LSTM models, and attention-augmented variants. The evaluation performed was subject dependent. Standardisation was performed before segmentation and the resulting windows were randomly divided without grouping by participant or trial. Thus, overlapping or otherwise related windows could appear in both the training and testing subsets. The metrics reported are an exploratory comparison specific to the protocol and do not predict performance on new participants. Figure 1 provides an overview of the processing pipeline.

Detailed descriptions of participant demographics, data acquisition protocols, preprocessing procedures, model configurations, and evaluation metrics are provided in the following subsections.

### Participants and demographics

The SDALLE database was created from a total of 9 healthy male subjects. Participants were between 12 and 39 years old (mean ± standard deviation, 26.6 ± 7.2 years). Mean height and body mass were 170.8 ± 12.9 cm and 80.3 ± 18.8 kg, respectively. All participants reported no history of disorders of musculoskeletal, neurological or gait disorders that could affect their locomotor performance. In this initial benchmarking study, the cohort was intentionally limited to healthy male participants to minimise biomechanical and neuromuscular variability. This controlled design was intended to increase internal consistency of the multimodal recordings and to allow a clearer comparison between the architectures evaluated. However, the lack of female participants, older adults, and clinical populations limits the generalisability across demographics and the direct extrapolation of the findings to broader real-world populations.

The following inclusion criteria were used to recruit participants: ability to independently perform locomotor activities without assistive devices, no known musculoskeletal or neurological disorders affecting gait, and no recent lower-limb injury or surgery within the previous six months. Exclusion criteria were any condition associated with impaired locomotion, balance disorders, chronic pain affecting movement, or inability to safely complete the experimental protocol. The sample size was primarily determined by practical and experimental constraints associated with high-resolution multimodal EMG–IMU acquisition, including extended setup time, sensor calibration requirements, signal-quality verification, and the duration of individual recording sessions. The study was designed as an initial controlled benchmarking and dataset-development effort to establish a reproducible multimodal locomotion dataset and to evaluate architectural behaviour under harmonised experimental conditions rather than population-scale clinical architectural consistency.

The study protocol involving human participants was reviewed and approved by the Scientific Research Ethical Committee, Faculty of Pharmacy, Egyptian Russian University (ERU), Cairo, Egypt (Reference No. ERUFE-MR-26-001; approval date: 13 May 2026). All experimental procedures involving human participants were conducted in accordance with the relevant ethical guidelines and regulations. Written informed consent was obtained from all adult participants prior to participation. For the minor participant included in the dataset, written parental consent was obtained from a legal guardian. Images included in this manuscript were cropped to remove identifiable facial features in accordance with journal anonymization guidelines. A summary of participant demographics is presented in Table 2.

**Table 2:** Participants demographics for the SDALLE dataset.

Subject	Age	Height (cm)	Weight (Kg)
1	39	164	77
2	30	184	89
3	23	173	77
4	26	162	65
5	23	179	100
6	29	168	66
7	28	175	96
8	29	187	105
9	12	145	48
Range (Mean ± SD)	26.6 ± 7.2	170.8 ± 12.9	80.3 ± 18.8

### Sensor instrumentation and setup

**Fig. 2:**
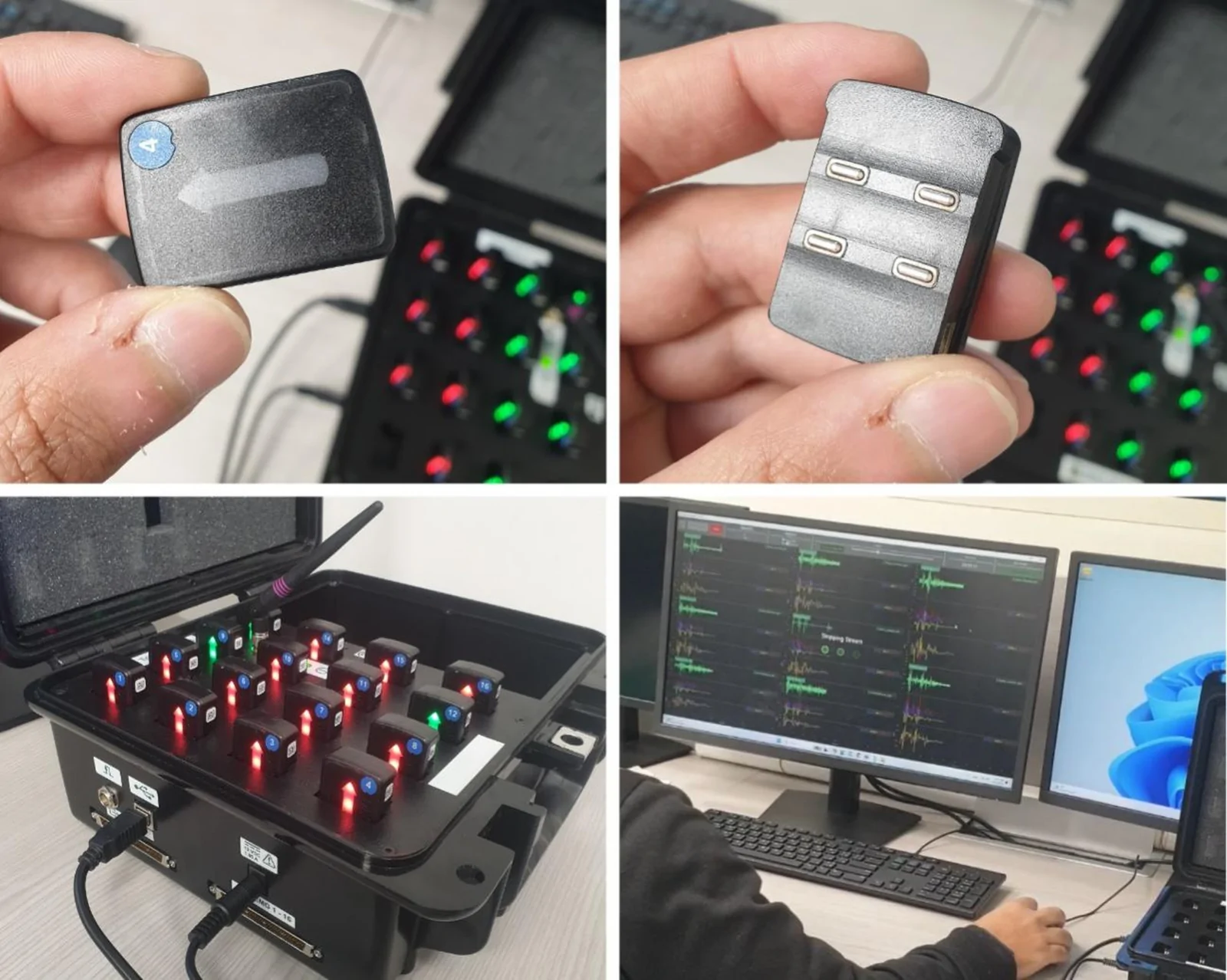
DELSYS Trigno Avanti wireless sensor system.

Surface electromyography and inertial measurement data were collected with the DELSYS Trigno wireless sensing platform (DELSYS Inc., Natick, MA, USA). Each Trigno sensor consists of a surface electromyography channel, combined with a nine-axis inertial measurement unit consisting of a triaxial accelerometer, gyroscope, and magnetometer. Although each Trigno sensor contains a nine-axis IMU, the exported dataset and subsequent analysis included only the triaxial accelerometer and triaxial gyroscope measurements. The 3 channels of magnetometer were not included in the preprocessing and classification. Therefore, seven channels have been considered for each of the eight sensors, one EMG and six inertial channels, for a total of 56 input channels. Such an integrated architecture allows an intrinsic synchronisation at hardware level between electromyography and inertial signals, thus avoiding the need of external synchronisation procedures (Fig. 2). To ensure repeatability between participants, multiple sensors were placed on the lower limbs according to standardised anatomical placement guidelines. The sensor data were wirelessly transmitted to a base station, which interfaced to a personal computer running Trigno Discover (v1.7.0). In the acquisition setup of the system configuration, the electromyography signals were sampled at 1259 Hz and the inertial signals at 148 Hz. The resulting configuration yielded high temporal resolution for the neuromuscular analysis, while maintaining stable low latency wireless communication. The IMU sampling rate was kept as in the original DELSYS Trigno acquisition configuration to preserve temporal fidelity during dynamic locomotor activities and maintain synchronised multimodal alignment with the EMG signals.

The compact form factor and untethered wireless design enabled participants to execute locomotor tasks under natural movement conditions without motion constraints. Sensors were attached with medical-grade adhesive interfaces and elastic fixation straps to reduce displacement artefacts during dynamic trials. The integrated multimodal sensing architecture reduced system complexity with respect to separate electromyography and inertial acquisition systems, guaranteed the temporal consistency between modalities, and delivered high-quality biosignals suitable for quantitative locomotion analysis and wearable human activity recognition applications.

### Sensor placement and calibration

**Fig. 3:**
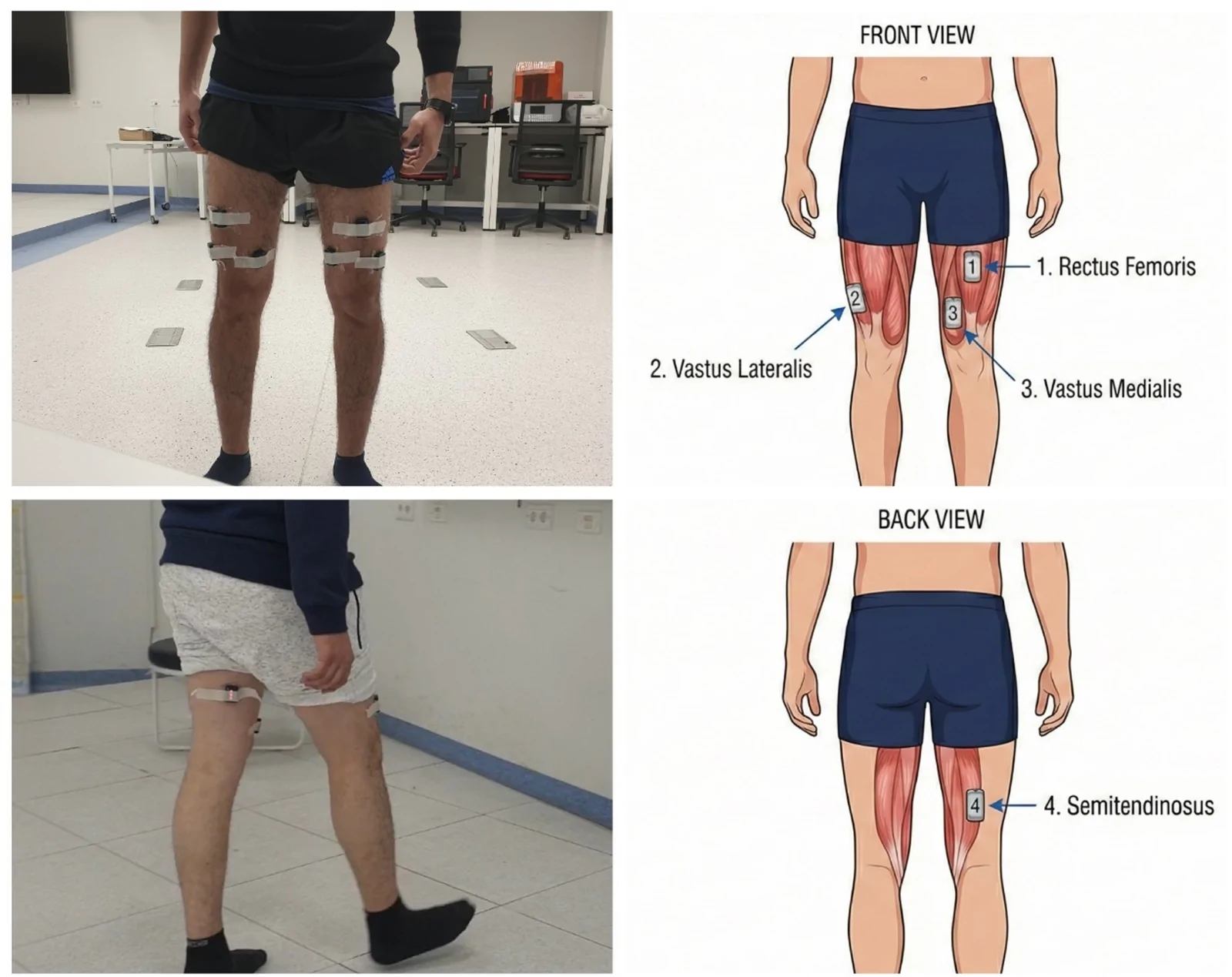
SDALLE dataset instrumentation setup showing bilateral sensor placement.

Accurate sensor placement was critical to ensure reliable electromyography acquisition and consistent inertial measurements across participants. In this study, eight Trigno sensors were placed bilaterally at four muscle sites per leg targeting the rectus femoris, vastus lateralis, vastus medialis and semitendinosus muscles of both lower limbs as shown in Fig. 3 and summarised in Table 3. Placement was performed according to standardised anatomical landmarks as defined by the SENIAM (Surface Electromyography for the Non-Invasive Assessment of Muscles) guidelines to increase repeatability and inter-subject consistency. Specifically, sensors were placed over the rectus femoris, vastus lateralis, vastus medialis, and semitendinosus muscles (Table 3). The sensor for the rectus femoris was placed on the anterior mid-line of the thigh, approximately half way between the anterior superior iliac spine and the superior border of the patella. The vastus lateralis sensor was positioned laterally on the mid-thigh and the vastus medialis sensor was positioned medially anterior on the thigh just proximal to the patella. The semitendinosus sensor was placed on the medial posterior thigh to record the hamstrings activation. The placement was bilateral and symmetric to guarantee comparable recordings between the left and right limbs. The skin at each placement site was shaved (when necessary), lightly abraded, and cleaned with alcohol wipes to reduce skin impedance and maximise electrode–skin contact before attaching the sensors. Medical-grade adhesive interfaces were used to secure the sensors, and elastic wraps reinforced the sensors to minimise motion artefacts during dynamic locomotor tasks. Following placement, each recording session was preceded by calibration and signal integrity checks per manufacturer specifications. Electromyography and inertial channels were inspected to ensure stable baselines, proper muscle activation responses during test contractions, and stable synchronisation across sensors. This verification procedure was conducted for each participant, and after any sensor repositioning, to guarantee consistent signal quality during data acquisition .

**Table 3:** Sensor placement locations applied bilaterally on each leg, yielding eight sensors in total.

Sensor Number	View	Muscle Name	Muscle Group	Sensor Location
1	Front Thigh	Rectus Femoris	Quadriceps	Middle of the thigh
2	Front Thigh	Vastus Lateralis	Quadriceps	Outer part of the thigh
3	Front Thigh	Vastus Medialis	Quadriceps	Inner part of the thigh
4	Back Thigh	Semitendinosus	Hamstrings	Medial posterior thigh

### Experimental procedure and activity protocol

**Fig. 4:**
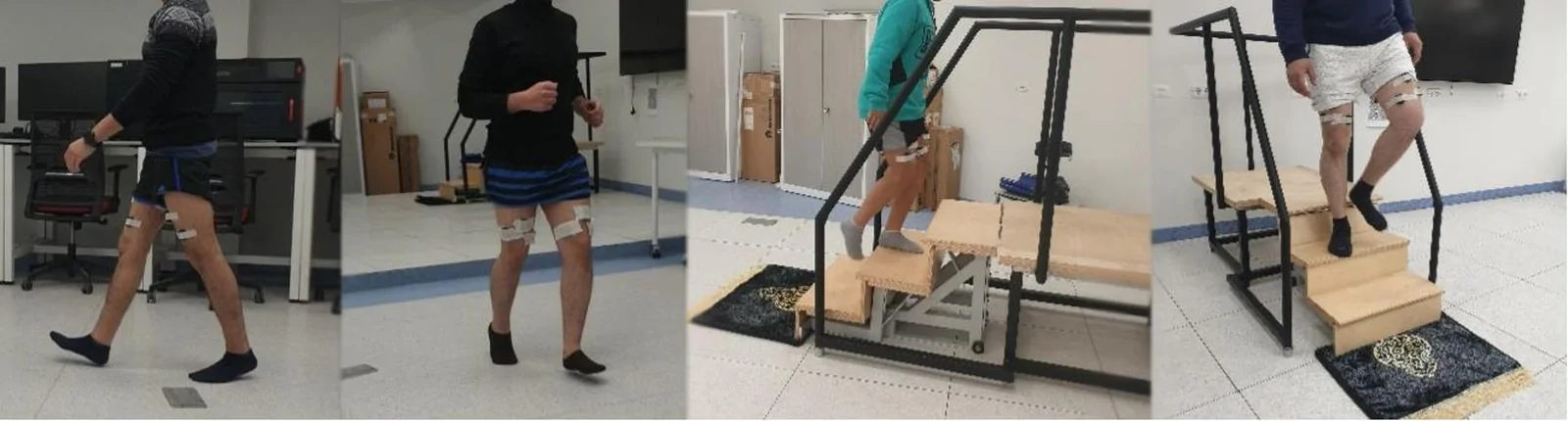
The four target activities (walking, jogging, ascending stairs, descending stairs).

The experimental protocol was aimed at capturing representative locomotor patterns relevant to wearable assistive systems and quantitative gait analysis. Each subject completed four standardised activities including walking, jogging, stair ascent and stair descent (Fig. 4). These activities were deliberately chosen as they represent basic lower-limb locomotor tasks that are commonly encountered in rehabilitation engineering, gait analysis, prosthetic control, and wearable assistive applications. The selected activities also span a range of neuromuscular and biomechanical challenges from steady-state locomotion to elevation-transition movements. After the sensors were placed and calibrated, the participants engaged in a 5-minute warm-up session, which consisted of light walking and gentle lower-limb stretching. This step helped to get familiarised with the instrumented setup and helped to reduce the chances of muscle strain during dynamic trials.

Walking and jogging trials were performed along a 20 m indoor corridor at a self-selected comfortable pace to preserve natural gait patterns. To ensure multiple gait cycles were recorded each trial participants were instructed to continuously walk or jog, turning at the end of the corridor as needed. Jogging was performed with the same spatial layout, but with fewer strides per pass due to the faster velocity.

Trials of stair ascent and descent were performed on a standardised indoor staircase with three steps (16 cm height, 30 cm depth per step) and an upper landing. The subjects were moving at a comfortable cadence and were asked to keep the same leading leg selection across repetitions to minimise intra-subject variability. The handrail was for you to hang on. The staircase configuration had three steps and was deliberately designed to offer controlled, repeatable and safe acquisition conditions within the available laboratory environment. Although this setup enabled consistent multimodal recordings across participants, it is a simplified approximation of real-world stair negotiation and may not fully capture the variability of daily-life stair locomotion.

Between trials a two-minute rest period was imposed to reduce fatigue and sustain signal quality. During these periods, sensor attachment and signal stability were briefly checked. Each recording session, including setup, warm-up, task execution, and rest periods, was approximately 50–80 min long. The standardised protocol guaranteed balanced and consistently labelled data acquisition over activities and participants. The experimental design controlled environmental and procedural variability, allowing for reliable signal segmentation and robust supervised learning analysis.

## Data acquisition and pre-processing

### Data recording and annotation procedure

**Fig. 5:**
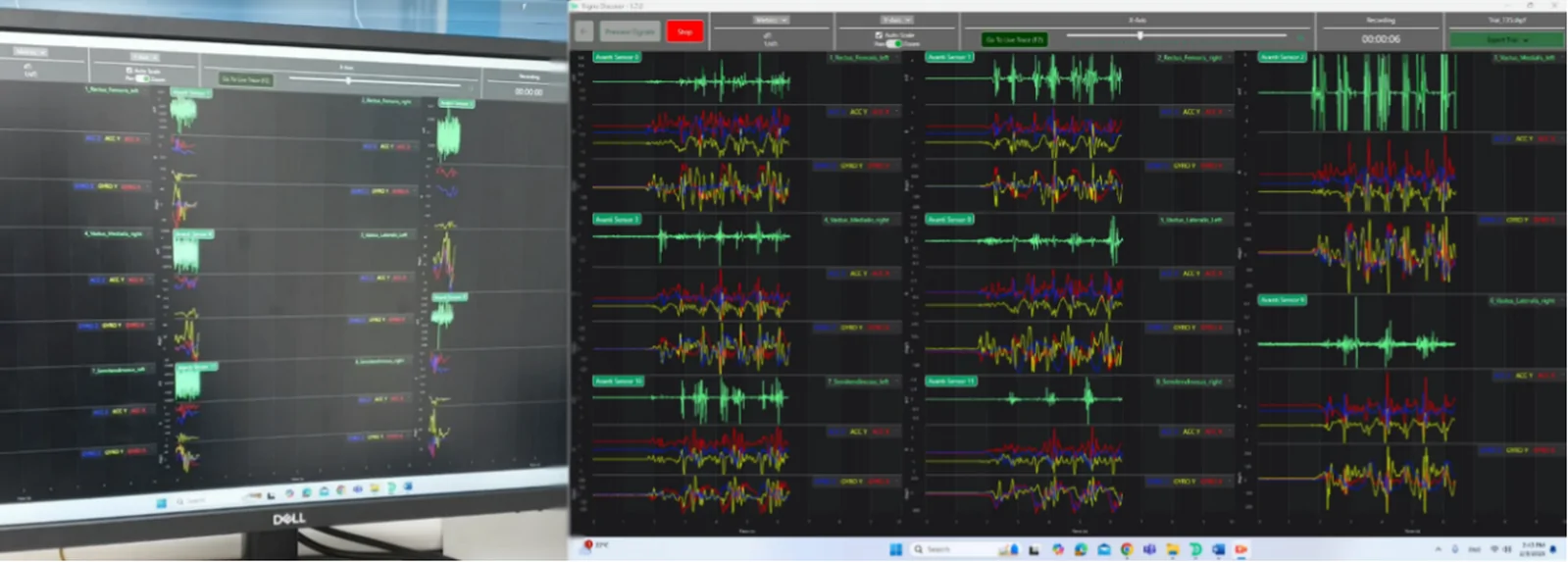
Data acquisition interface of DELSYS Trigno discover software.

All sensor data were acquired with the DELSYS Trigno wireless system interfaced with the Trigno Discover software (v1.7.0). The acquisition interface was configured to acquire simultaneously the electromyography and inertial channels at the maximum sampling rates allowed by the experimental set-up. The electromyography signals were recorded at 1259 Hz while inertial signals (triaxial acceleration and angular velocity) were recorded at 148 Hz. These acquisition frequencies are consistent with internal hardware specifications within the sensing platform and were chosen to optimise temporal fidelity. In each trial, the acquisition interface allowed also to monitor in real-time all the active channels (Fig. 5), in order to check on-site the integrity of the signal (e.g. presence of electromyographic activation bursts and peaks related to inertial impacts). The electromyography and inertial signals were inherently synchronised at the time of acquisition because all channels were sharing a common timestamp reference within the Trigno system, thus avoiding the need for external triggering or post-hoc temporal correction.

Each trial was exported in Comma-Separated Values (CSV) format for compatibility with Python-based preprocessing and analysis pipelines. Recordings included data from eight sensors (four per leg), each providing one electromyography channel and six inertial channels (three axes of acceleration and three axes of angular velocity). Each file was enriched with an extra column containing the activity label.

Due to the disparity in sampling frequencies, electromyography signals contained approximately 8.5 times more samples per second than inertial signals. Although temporal alignment was preserved through shared timestamps, the difference in sampling density required harmonization during preprocessing, as described in Sect.  3.3. For each trial, detailed acquisition logs were kept indicating participant identifier, type of activity, repetition number and any anomalies (e.g., missteps or sensor displacement). Trials containing artefacts or interruptions were discarded and were repeated to ensure consistency in the dataset. At the end of data collection, each participant provided the same number of recordings for each activity. In summary, the recording procedure provided high-resolution, time-synchronized multimodal data that are suitable for structured preprocessing and supervised learning.

### Dataset structure and storage format

Following acquisition, all recordings were organized into a unified dataset structure compatible with supervised learning workflows. Each sample was labelled with a numeric activity label (0: walking, 1: jogging, 2: stair ascent, 3: stair descent) by adding an ActivityLabel column. After sampling-rate alignment (Sect.  3.3), trials corresponding to the same activity were concatenated at the participant level, and participant-level datasets were subsequently merged into a master dataset. Each row corresponds to a timestamped observation which includes synchronised electromyography and inertial measurements along with the associated activity label. Participant identity was retained in the acquisition records but was not used as a grouping variable when the modeling data were partitioned; consequently, data from the same participant and trial could contribute windows to both training and testing subsets.

The dataset was primarily stored in the CSV format to ensure architectural consistency and interoperability across platforms. The SDALLE dataset, comprising raw recordings, processed datasets, and preprocessing scripts, was made publicly available on Zenodo and assigned a digital object identifier^31^ to foster reproducibility and open science practices. Documentation is provided describing the sensor configuration, file structure and use.

### Data synchronization and sampling rate alignment

**Fig. 6:**
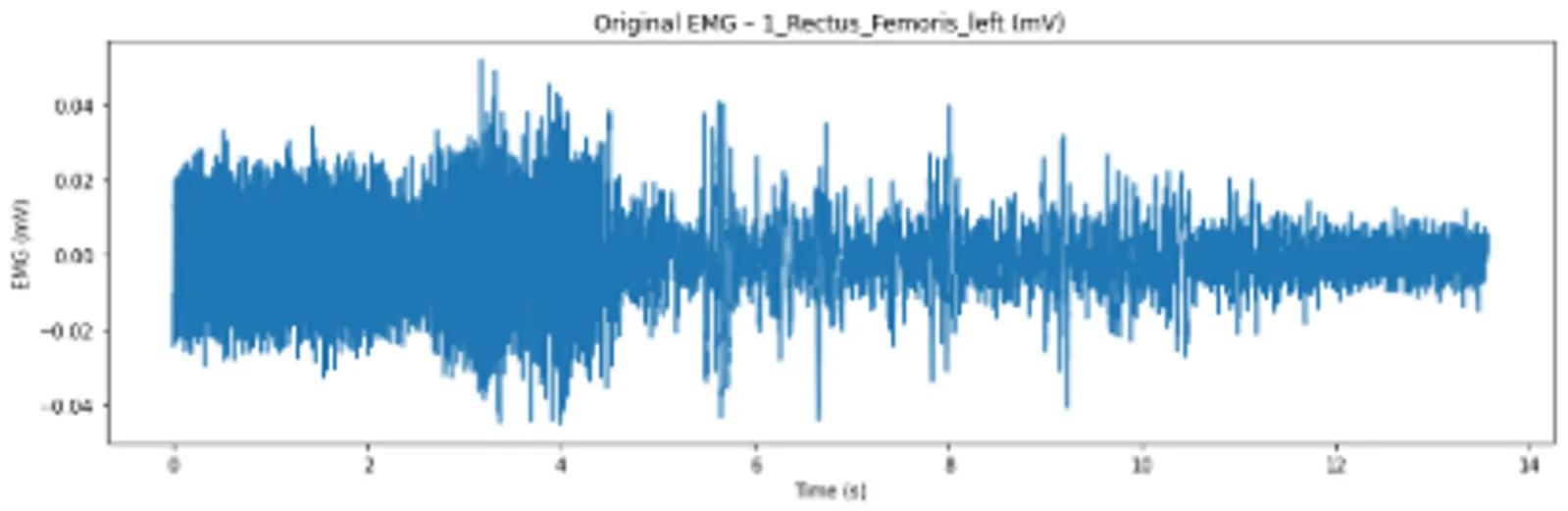
The original high-resolution EMG sampled at 1259 Hz during walking.

**Fig. 7:**
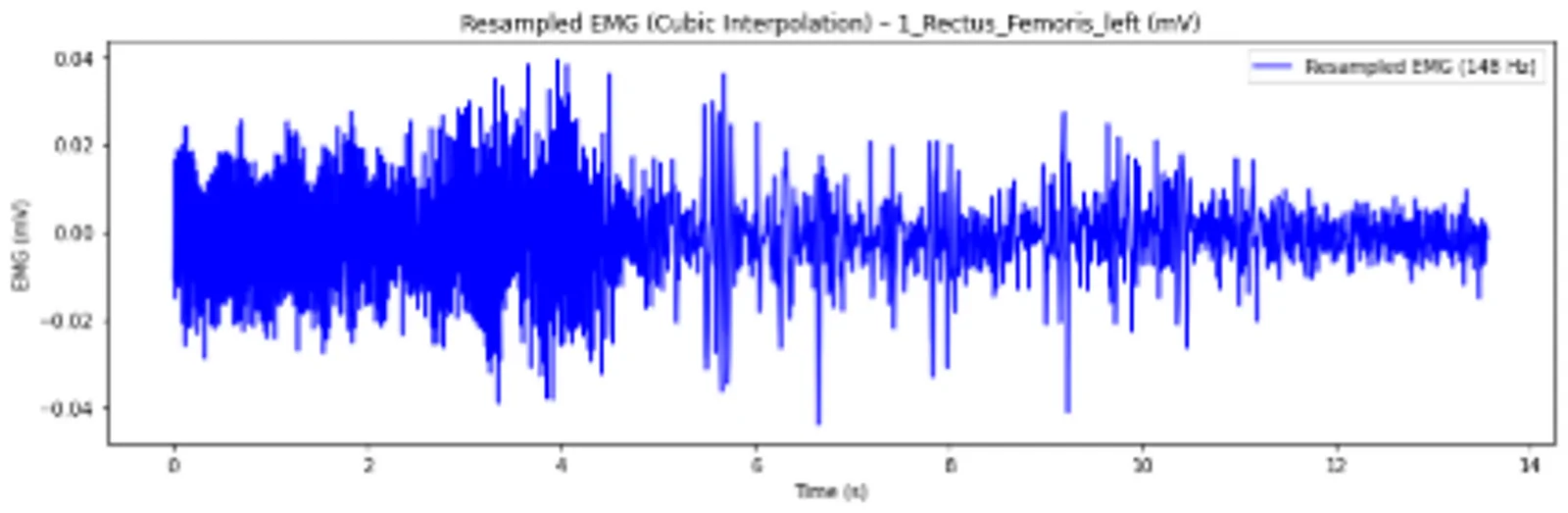
Downsampled EMG Signal After Cubic Interpolation to IMU Sampling Rate 148 Hz.

**Fig. 8:**
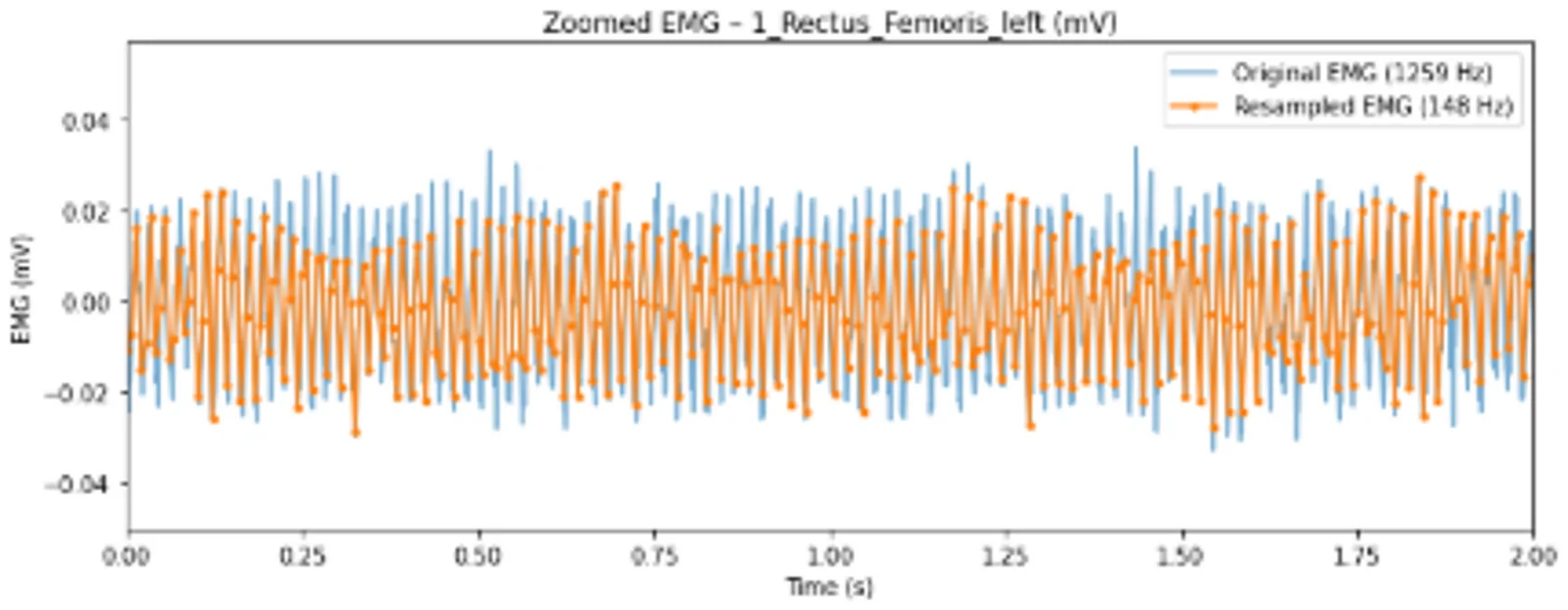
Zoomed EMG Segment Showing Original (1259 Hz) vs. Resampled (148 Hz) Signals.

A major challenge of the pre-processing was the difference in sampling rates of the electromyography (1259 Hz) and inertial (148 Hz) sensors. Both modalities were synchronised in time at acquisition, but joint analysis required a uniform sampling density. Hence, electromyography signals were downsampled to 148 Hz by cubic interpolation. The reason for selecting this approach is to preserve signal continuity and amplitude envelope characteristics while minimising artefacts associated with simpler linear interpolation methods. The downsampling was mainly performed to enable the temporally synchronised multimodal EMG-IMU fusion in a unified deep learning framework for recognition of locomotor activities. The aim was not to preserve the whole high frequency spectrum of EMG but to preserve the activation timing, envelope features and multimodal temporal relations relevant to Human Activity Recognition. Raw and resampled electromyography signals were visually compared to verify preservation of physiologically meaningful information. Figure 6 shows an example of the original high resolution signal and Fig. 7 shows the corresponding downsampled version. Figure 8 magnifies a two second segment and demonstrates that the timing of the activation, the burst structure and the gait-cycle patterns were maintained after resampling. The downsampled signal looks smoother as one would expect that high frequency components will be lost, but the relevant activation dynamics are not distorted.

Major patterns of activation and temporal structure were preserved with cubic interpolation but inevitably downsampling attenuated higher frequency EMG components. Therefore, the results should be interpreted as part of synchronised multimodal locomotor HAR rather than detailed EMG spectral analysis. All sensors were synchronised to a common base station, thus eliminating drift across trials between sensors. The aligned dataset provided a time-consistent multimodal signal representation for segmentation and activity recognition based on deep learning. In order to preserve the temporal continuity and to improve the representation of the transitional locomotor dynamics contained in sequential EMG-IMU signals, we performed sliding-window segmentation with 75% overlap.

For the SDALLE dataset, the aligned EMG-IMU signals were segmented with a window length of 16 timesteps and with a stride of 4 timesteps or 75% overlap. At the aligned sampling rate of 148 Hz, each window spanned approximately 0.108 s and each stride approximately 0.027 s. Thus, adjacent windows shared 12 of 16 samples. For ENABL3S, a window length of 128-time steps and a stride of 32-time steps were used, which is also equivalent to 75% overlap. Each ENABL3S window was approximately 0.256 s long with a stride of 0.064 s at 500 Hz, with 96 of the 128 samples shared between adjacent windows. This overlap can lead to very similar windows being assigned to different subsets, as the random train/test splitting described in Sect.  5.2 was done after segmentation. Hence, statistical independence of the training and test windows is not guaranteed. Table 4 summarises the segmentation parameters.

**Table 4:** Sliding-window segmentation parameters used for each dataset.

Dataset	Effective sampling rate	Window length	Window duration	Stride	Stride duration	Overlap
SDALLE	148 Hz	16 samples	0.108 s	4 samples	0.027 s	75%
ENABL3S	500 Hz	128 samples	0.256 s	32 samples	0.064 s	75%

Feature-wise z-score standardization was performed separately for each dataset and independently for each sensor channel according to:

$$\:{Z}_{ij}=\left({x}_{ij}-\:{\mu\:}_{j}\right)/\:{\sigma\:}_{j}$$

Where $\:{x}_{ij}$ is observation * i* of channel * j*, and $\:{\mu\:}_{j}$and $\:{\sigma\:}_{j}$ are the mean and standard deviation of that channel, respectively. The standardized values are dimensionless, with approximately zero mean and unit variance. In the implemented pipeline, these statistics were calculated from the complete merged dataset before window segmentation and the randomized train/test partition; therefore, they were not estimated from the training subset alone.

## Dataset enhancement using generative adversarial networks

To mitigate class imbalance and moderate dataset size limitations in SDALLE, a generative data augmentation strategy was adopted to synthesize additional electromyography–inertial time-series samples. Before augmentation, the walking class made up about 36% of the dataset, while jogging, stair ascent and stair descent were relatively under-represented, resulting in moderate class skew. We used a time-series Wasserstein Generative Adversarial Network with Gradient Penalty (WGAN-GP) to model the joint distribution of multivariate electromyography–inertial signals. The WGAN-GP framework was chosen because of improved training stability and less mode collapse compared to standard adversarial formulations, which makes it suitable for continuous-valued sequential data^32,33^. Both the generator and critic were implemented using Long Short-Term Memory architectures to capture temporal dependencies intrinsic to the locomotor dynamics.

**Fig. 9:**
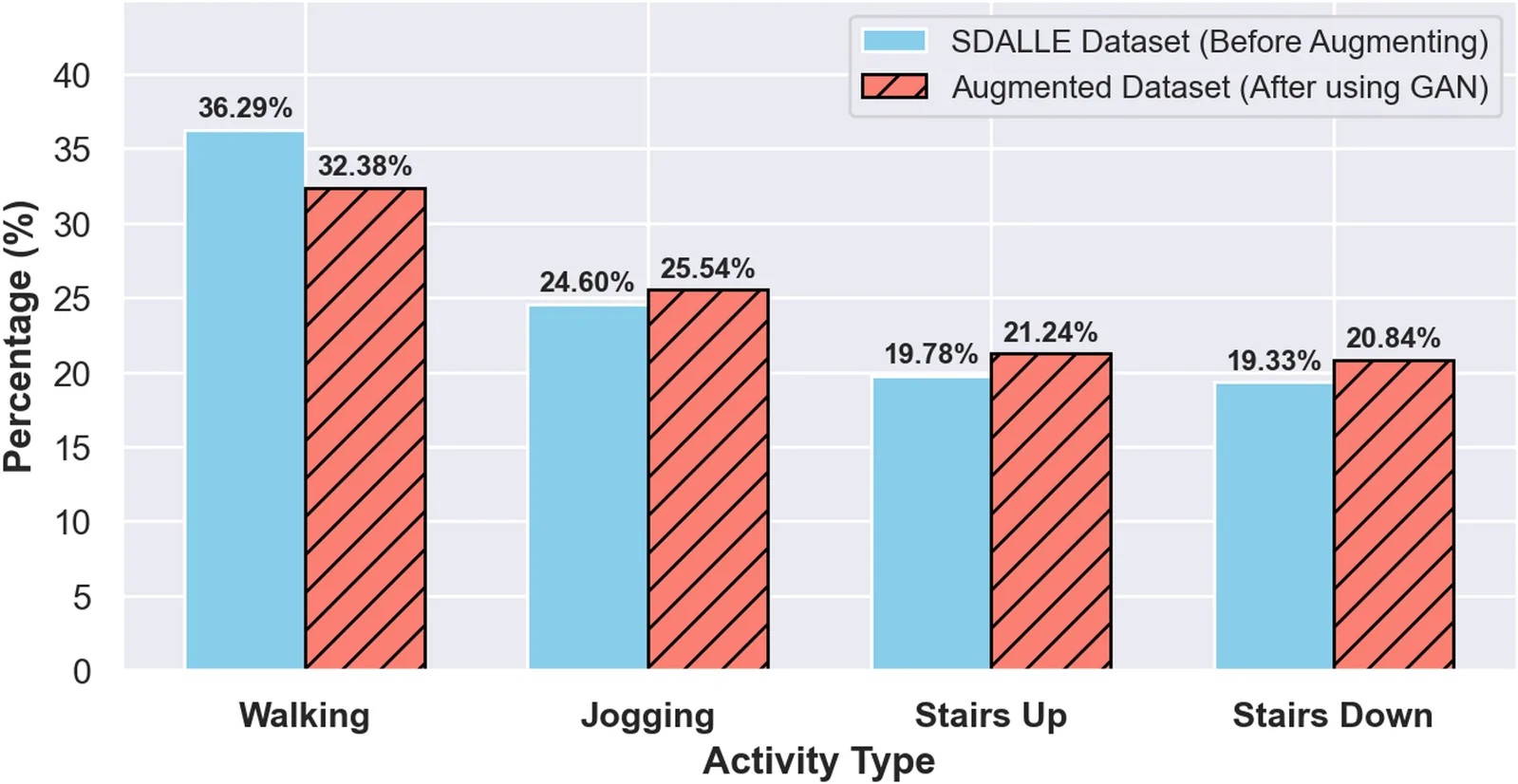
Activity distribution before and after GAN-based augmentation.

Synthetic data were generated independent of participant-activity files, using 96-timestep windows with 75% overlap. Continuous sequences were obtained by overlap-add of generated windows, thus minimising boundary discontinuities. Augmentation was applied to jogging, stair ascent and stair descent; walking was unchanged. For each of the nine participants, 2,000 synthetic time-series rows were added for each of the three minority activities, producing approximately 54,000 added rows and increasing the dataset from 446,861 to 500,861 rows (approximately 12%). Thus, the walking proportion was reduced from 36.3% to 32.4% (see Fig. 9). Augmentation was done before merging, standardisation, segmentation into classifier windows and random splitting of the participant files. Hence GAN training and preprocessing were not restricted to a participant-independent training fold. Thus the resulting before/after comparison is exploratory and depends on the data and cannot be interpreted as evidence of the performance on unseen participants.

## Experimental design

### Deep learning architectures

**Fig. 10:**
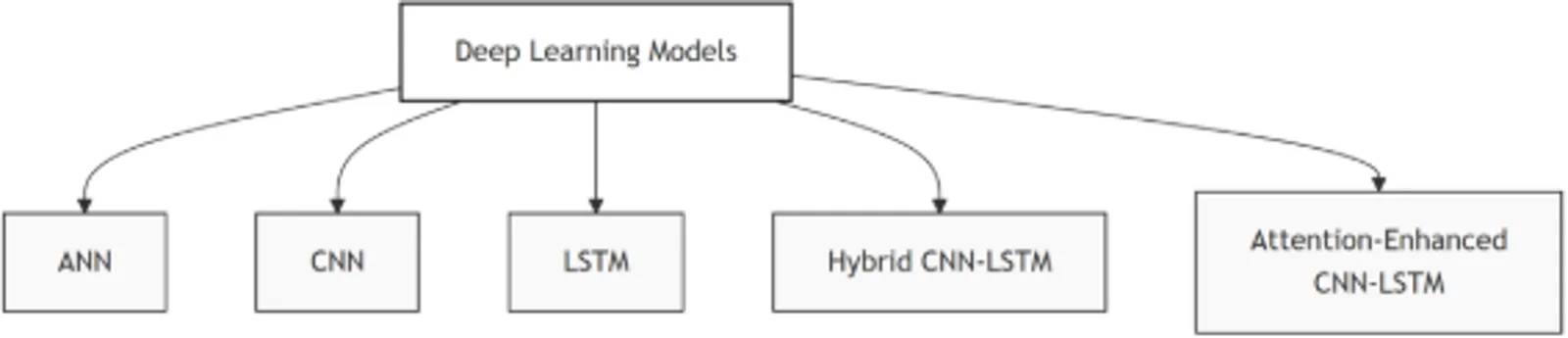
Deep learning architectures evaluated in this study.

The evaluated architectures and their associated hyperparameters were selected from commonly used configurations in Human Activity Recognition and multivariate time-series learning, as well as from preliminary empirical testing for stable convergence. The benchmark included feedforward, convolutional, recurrent, hybrid spatiotemporal and attention-enhanced models. The model families are summarised in Fig. 10. To minimise procedural variation all architectures shared the same preprocessing and optimisation pipeline. However, this standardisation does not make the test set participant independent, since the highly overlapping windows were randomly partitioned, and different architectures may benefit differentially from the repeated information. Thus, the observed model ranking is conditional on the window-level protocol implemented and is not reported as a participant-independent comparison.

#### Artificial neural networks (ANNs)

As a baseline architecture, a fully connected feedforward network was implemented to give a reference model without explicit temporal or spatial inductive biases. The network architecture consisted of four hidden dense layers with decreasing dimension (256, 128, 64 and 32 units) with rectified linear unit (ReLU) activation functions. Feedforward networks expect two-dimensional input tensors of shape (samples, features). The segmented EMG–IMU windows were therefore reshaped from their original three-dimensional structure (samples × timesteps × channels) into flattened feature vectors before training. While the ANN is able to learn nonlinear feature interactions, it doesn’t explicitly model the temporal dependencies or the local cross-channel correlations that are intrinsic to sequential sensor data. It is thus a static feature-combination baseline for quantitative comparison to spatio-temporal specific architectures such as convolutional, recurrent and hybrid architectures.

#### Convolutional neural networks (CNNs)

**Fig. 11:**
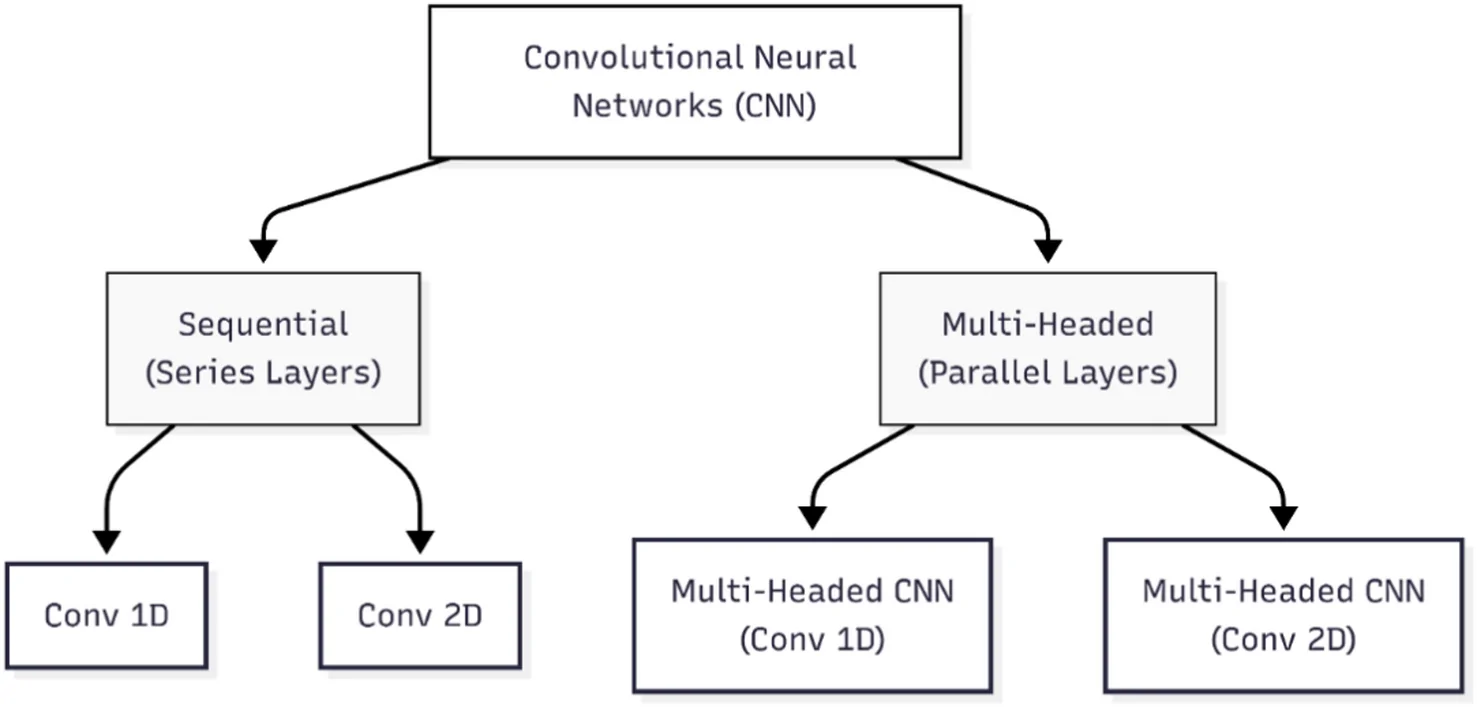
Evaluated sequential and parallel CNN architectures.

Convolutional Neural Networks were evaluated to explore their ability to extract localised spatiotemporal features from segmented EMG-IMU windows. We investigated other feature extraction approaches by using one-dimensional (Conv1D) and two-dimensional (Conv2D) variants and studied their performance in sequential and multi-headed configurations. Figure 11 summarises the CNN architectures being evaluated. Table 5 summarises the main layer configuration of the evaluated CNN architectures.

The CNN architectures evaluated in this study were adopted from commonly used deep learning paradigms in time-series Human Activity Recognition and implemented within a unified pipeline to maintain consistent implementation conditions across datasets and model families. This standardization does not establish participant-independent comparability.

**Table 5:** Summary of evaluated CNN architectures.

Component	Conv1D CNN	Conv2D CNN	Multi-Head CNN
Input Representation	Time-series	Pseudo-image	Parallel time-series branches
Conv Layer 1	64 filters, kernel = 3	64 filters, 3 × 3	Kernels = 3,5,11
Conv Layer 2	128 filters, kernel = 3	128 filters, 3 × 3	Parallel feature extraction
Conv Layer 3	265 filters, kernel = 3	265 filters, 3 × 3	Parallel feature extraction
Dense Layers	100, 64	100, 64	100, 64
Activation	ReLU	ReLU	ReLU
Output Layer	SoftMax	SoftMax	SoftMax

The standalone sequential Conv1D and Conv2D models consisted each of three stacked convolutional layers followed by two fully connected layers of 100 and 64 units. In contrast, the multi-headed CNN (Fig. 1) consisted of three parallel branches with each branch having a single convolutional layer with 64 filters and kernel sizes 3, 5 and 11, respectively. These branches were concatenated prior to the fully connected classification layers. For the CNN-LSTM variants we used the architecture specific convolutional configurations described in Sect.  5.1.4.

##### CNN (Conv1D)

The Conv1D architecture applies 1D convolutions along the temporal axis of each segmented window with inputs shape of (time_steps × sensor_channels). This formulation allows the model to capture local temporal patterns while maintaining inter-channel structure across EMG-IMU signals. Hierarchical temporal features were extracted by stacking convolutional layers with increasing effective receptive field. The design captures short-term waveform characteristics and mid-range temporal dependencies, without explicitly modelling long-range sequential dynamics.

##### CNN (Conv2D)

**Fig. 12:**
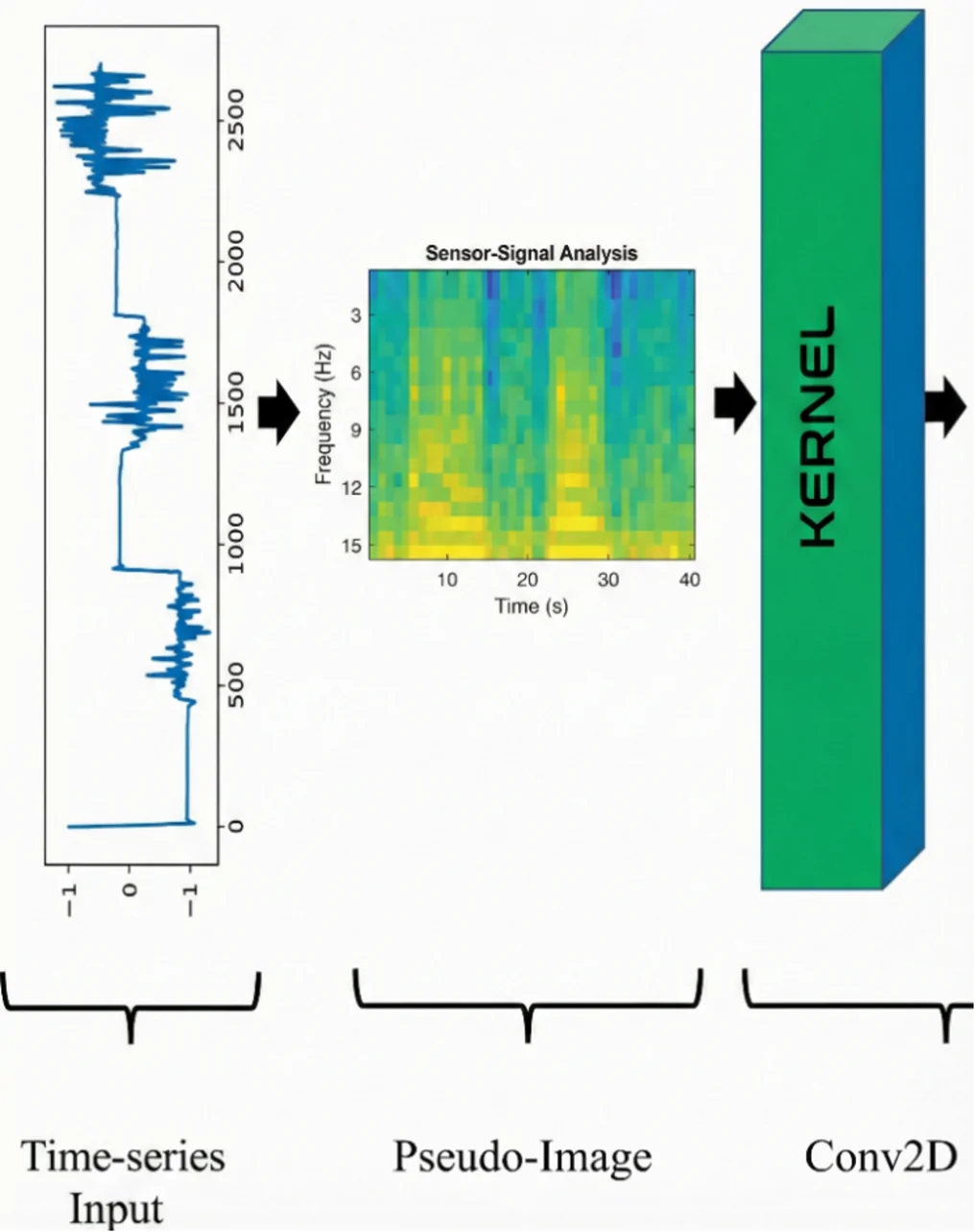
Transformation of time-series windows into two-dimensional pseudo-image representations for Conv2D processing.

A variant of Conv2D was implemented to study if the joint time-channel interactions could be modelled more efficiently. Each segmented window was reshaped into a two-dimensional representation (time_steps × sensor_channels), treated as a single-channel pseudo-image (Fig. 12).

In this formulation the convolutional kernels interact across the temporal and cross-channel dimensions to allow the network to learn local patterns across neighbouring time steps and across multiple sensor channels. Furthermore, the kernels of Conv2D learn structured inter-channel dependencies in local temporal neighbourhoods, in contrast to Conv1D filtering along the temporal axis only.

##### Multi-headed CNN architectures

**Fig. 13:**
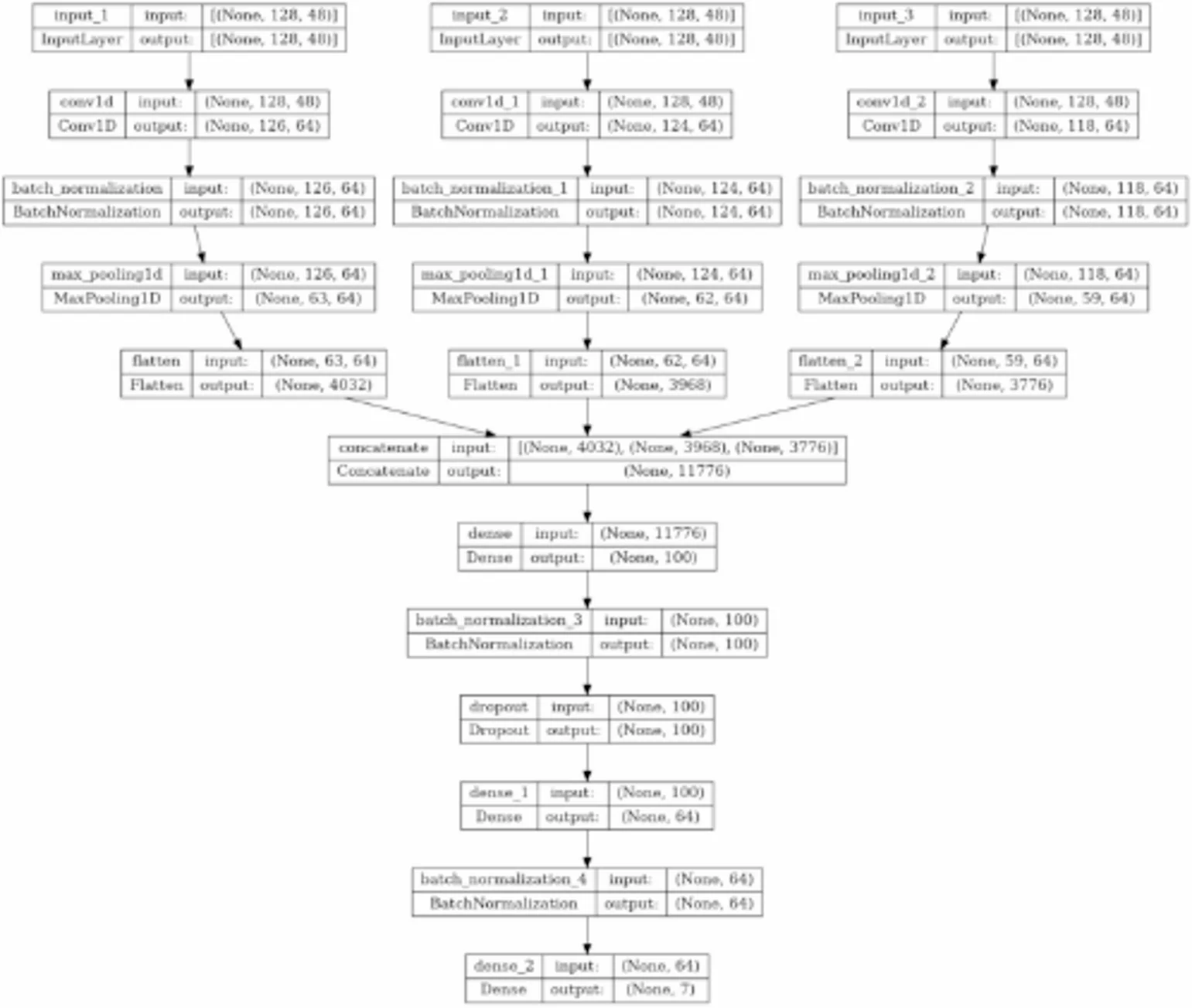
Multi-headed CNN architecture with parallel convolutional branches.

Besides the sequential CNN configurations, multi-headed CNN configurations were also studied to capture features at multiple temporal scales. As shown in Fig. 13, we applied parallel convolution branches with different kernel sizes (e.g., 3, 5 and 11) on the same input window. Smaller kernels targeted fine-grained temporal fluctuations, while larger kernels captured broader signal trends. The outputs of the parallel branches were concatenated and then fed into fully connected layers, which allowed to integrate multi-scale feature representations in a unified classification framework.

#### Long short-term memory (LSTM) networks

Long Short-Term Memory (LSTM) networks were utilised to explicitly model temporal dependencies within the sequential EMG–IMU data. LSTMs can learn long-range temporal relationships while avoiding vanishing gradient effects, making LSTMs especially suitable for multivariate time-series classification, due to the gated memory mechanism of LSTMs. We used a stacked LSTM architecture with three recurrent layers of 512, 256 and 128 hidden units respectively. The recurrent layers were followed by 2 fully connected layers (100 and 64 units, ReLU activation) and a softmax layer for multi-class activity classification. Input sequences were structured as (time_steps, features), maintaining the temporal ordering and multichannel structure of the segmented sensor windows. In contrast to feedforward and convolutional models, the LSTM architecture processes the sequence recurrently, allowing the network to learn temporal dynamics across gait cycles and activity transitions, without the need for explicit window-level temporal aggregation. We use this configuration as a purely recurrent modelling baseline against which we compare the convolutional and hybrid spatiotemporal architectures considered in this work.

#### Hybrid CNN–LSTM architectures

**Fig. 14:**
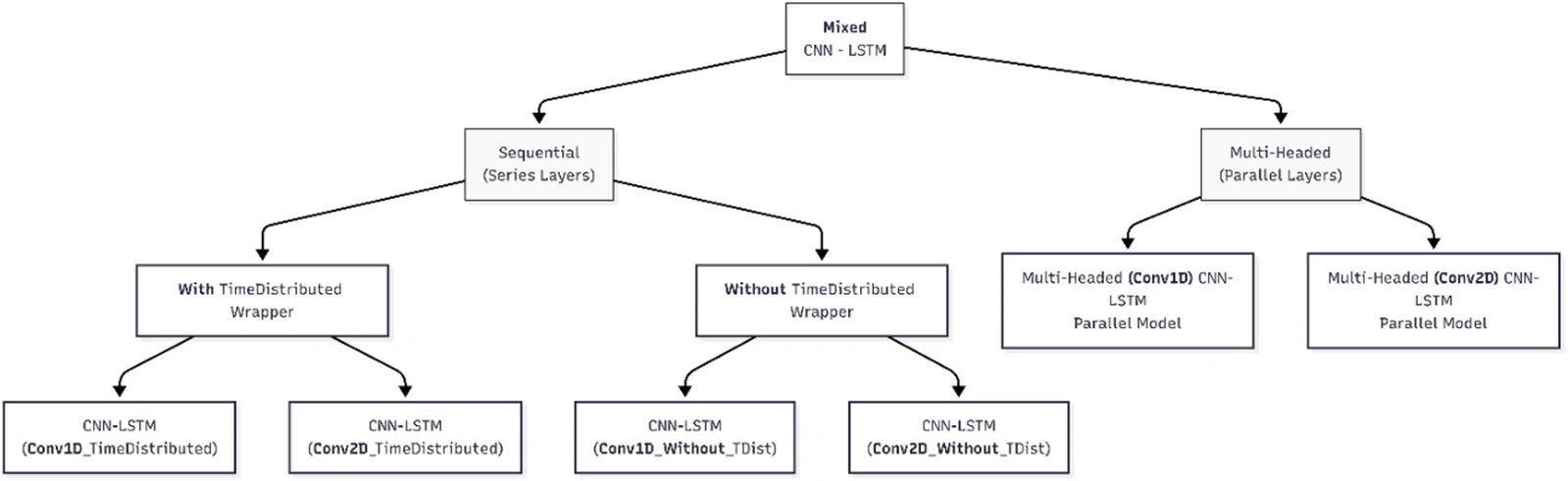
Evaluated hybrid CNN–LSTM architectures.

The hybrid CNN–LSTM configurations evaluated in this study are based on established spatiotemporal deep learning designs reported in the literature of wearable HAR, and were implemented under harmonised experimental settings for comparative evaluation. Hybrid CNN–LSTM architectures were designed to combine convolutional feature extraction with recurrent temporal modelling in a unified spatiotemporal framework. Convolutional layers learn local time-channel patterns from segmented EMG-IMU windows, and Long Short-Term Memory layers capture the temporal evolution of the learned representations. The two hybrid configurations evaluated are shown in Fig. 14.

**(i) Sequential (Series) CNN–LSTM**.

In the sequential setting, the recurrent layers follow the convolutional layers. The convolutional blocks produce feature maps, which are fed as input sequences to the LSTM layers so that the recurrent modelling of the temporally ordered convolutional representations can be realised. This architecture allows processing the local spatial descriptors obtained from the convolutional filters, to capture the dynamic evolution over the gait cycles. There are two ways we considered to interface convolutional outputs to recurrent inputs:

- *Time Distributed variant* Convolutional operations were applied using a TimeDistributed wrapper, ensuring that temporal ordering was preserved explicitly. This approach generates feature sequences directly compatible with LSTM input format while maintaining alignment between convolutional features and original time steps.
- *Reshaped variant*: Convolutional feature maps were manually reshaped into tensors of shape (samples, time_steps, features) before being passed to LSTM layers. Although this method offers flexibility in feature restructuring, careful reshaping was required to maintain temporal coherence.

Both variants were evaluated to examine whether explicit preservation of temporal structure during convolution influences downstream sequence modeling performance.

**(ii) Multi-Headed (Parallel) CNN–LSTM**.

In the parallel setting, both the convolutional and the recurrent branch processed the same input window, and the outputs of the two branches were concatenated before classification. The convolutional branch extracted localised features, and the LSTM branch modelled sequential dependencies. A consistent backbone was maintained to reduce implementation variation across the hybrid configurations; The convolutional block consisted of 2 layers with 64 and 128 filters and 2 LSTM layers with 256 and 128 units. The classifier consisted of two fully connected layers (100 and 64 units, ReLU activation) and a SoftMax output. These standardised design choices do not eliminate the limitations coming from the randomised split of the window into windows. The main configurations of CNN-LSTM and attention based are summarised in Table 6.

**Table 6:** Summary of evaluated CNN–LSTM and attention-based architectures.

Component	CNN–LSTM	Multi-head CNN–LSTM	Attention CNN–LSTM
CNN Layers	64, 128 filters	Parallel CNN branches	64, 128 filters
LSTM Units	256, 128	256, 128	256, 128
Attention Module	No	No	Yes
Dense Layers	100, 64	100, 64	100, 64
Output Layer	SoftMax	SoftMax	SoftMax

#### Attention-enhanced CNN–LSTM architectures

**Fig. 15:**
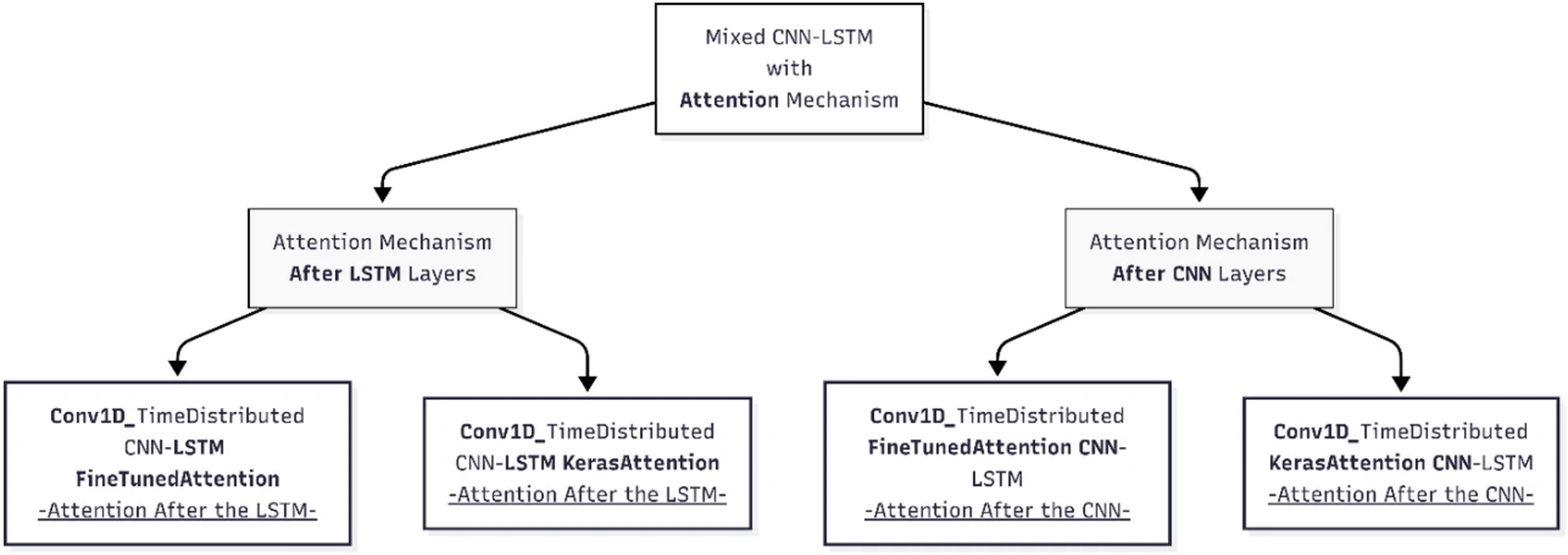
Hybrid CNN–LSTM architectures with attention mechanisms.

Attention-enhanced CNN–LSTM models enhance the hybrid spatiotemporal framework by incorporating temporal attention mechanisms which adaptively weight informative time steps within each input sequence. Conventional CNN-LSTM architectures capture local spatiotemporal features and model their sequential evolution, but the inclusion of attention provides a learnable weighting function that highlights activity-discriminative temporal regions. As shown in Fig. 15, attention mechanisms were incorporated at two different stages of the hybrid pipeline to evaluate their relative impact on multimodal EMG–IMU activity recognition. We experimented with attention mechanisms at different stages of integration to determine whether temporal emphasis works better in the convolutional feature extraction or recurrent temporal modelling. The attention modules were implemented as standard sequence-weighting approaches commonly used in the deep learning literature, rather than being new attention formulations.

In the first configuration, attention was used after the stacked LSTM layers. In the post-LSTM setting, attention was applied on the sequence of the hidden state vectors to generate normalised temporal importance weights to create a weighted context vector summarising the sequence. The second configuration included the attention mechanism over the convolutional layers (post CNN) and enabled the adaptive weights of the convolutional feature maps before the recurrent temporal modelling. This design enables to highlight salient feature activations before sequence-level modelling. By evaluating both placements, it is possible to isolate whether temporal emphasis is more useful at the feature-extraction or at the recurrent modelling stage.

##### Fine-tuned attention

A custom temporal attention layer was implemented in TensorFlow/Keras, and appended after the stacked LSTM layers. The hidden state vectors were projected through a learnable transformation, and then passed through nonlinear activation, and SoftMax normalisation across time steps to produce attention weights. Finally, the last context vector was computed as weighted sum of LSTM outputs. This formulation allows the joint optimisation of the attention parameters with the CNN–LSTM backbone. In this way, the network could adapt the temporal weighting to the multimodal EMG–IMU characteristics of the dataset. The fine-tuned version therefore makes the modelling more flexible and allows an explicit control of emphasis in time.

##### Keras-based attention

We also experimented with the built-in Keras attention layers to provide a standard baseline. These layers used LSTM outputs to compute attention scores and produce weighted temporal representations with no custom weighting formulation. The Keras-based model thus formed a reference for the custom fine-tuned formulation, and the convolutional and recurrent backbone was unchanged in all variants. Such controlled implementation reduced architectural variation but does not disentangle attention effects from the effect of the non-independent window-level split. Recognition and class-level differences are interpreted only within the implemented protocol.

### Training setup

For hyperparameter optimisation we did not perform exhaustive architecture specific optimisation but rather focus on comparative benchmarking under harmonised optimisation settings. The experiments were performed on a workstation with an AMD Ryzen 9 5950X CPU, 32 GB of RAM, and an NVIDIA RTX 3090 GPU with 24 GB of memory. The software environment was Ubuntu 22.04, Python 3.11, TensorFlow 2.15, CUDA 12.1, and cuDNN 8.9.7. All architectures were trained with Adam (learning rate = 0.001) for 30 epochs with batch size of 64, sparse categorical cross-entropy loss, ReLU activations in convolutional layers and Glorot-uniform weight initialisation. Samples in each input window were preserved in temporal order. Feature-wise z-score standardisation was fitted on the complete merged dataset before window segmentation individually for each dataset. Then, the segmented windows were randomly split into 70% training and 30% testing subsets (shuffle enabled; random seed = 42) without grouping by participant or trial. The GAN-generated data were also prepared before this final partition for the augmented SDALLE analysis. As a result, training and testing subsets may contain overlapping windows that share raw samples and windows from the same participant and trial. The full-dataset standardisation uses information from observations that are later assigned to the test subset. Such factors may lead to optimistic estimates of tests. They may affect models differently. Thus, all reported results are exploratory, participant-dependent window-level measurements and should not be taken as evidence of generalisation to unseen participants. Table 7 reports the complexity of the architecture and the computational cost of the evaluated models.

**Table 7:** Architectural Complexity and computational cost of evaluated models.

Model	Trainable parameters (Approx.)	Training Time (30 epochs)	Testing time
ANN	~ 0.45 M	Lowest	Very Low
CNN Conv1D	~ 0.85 M	Low	Low
CNN Conv2D	~ 1.10 M	Low–Moderate	Low
Multi-Head CNN	~ 1.45 M	Moderate	Low
LSTM	~ 3.80 M	Moderate–High	Moderate
CNN–LSTM (Sequential)	~ 4.20 M	High	Moderate
CNN–LSTM (TimeDistributed)	~ 4.50 M	High	Moderate
Multi-Head CNN–LSTM	~ 5.10 M	High	Moderate
CNN–LSTM + Keras Attention	~ 5.40 M	Very High	Moderate
CNN–LSTM + Fine-Tuned Attention	~ 5.70 M	Highest	Moderate

### Evaluation metrics

Standard multi-class classification metrics were used to summarise model performance. Overall accuracy was the main measure while precision, recall and F1-score characterised class-sensitive behaviour in case of residual imbalance. These metrics were computed over the randomised 30% window-level test subset described in Sect.  5.2. They thus measure subject-dependent window classification in the implemented pipeline, and do not predict performance for participants not included in training. SDALLE and ENABL3S comparisons are descriptive and protocol specific, not evidence of cross subject generalisation.

## Results

### Subject-dependent window-level performance on the SDALLE dataset

**Fig. 16:**
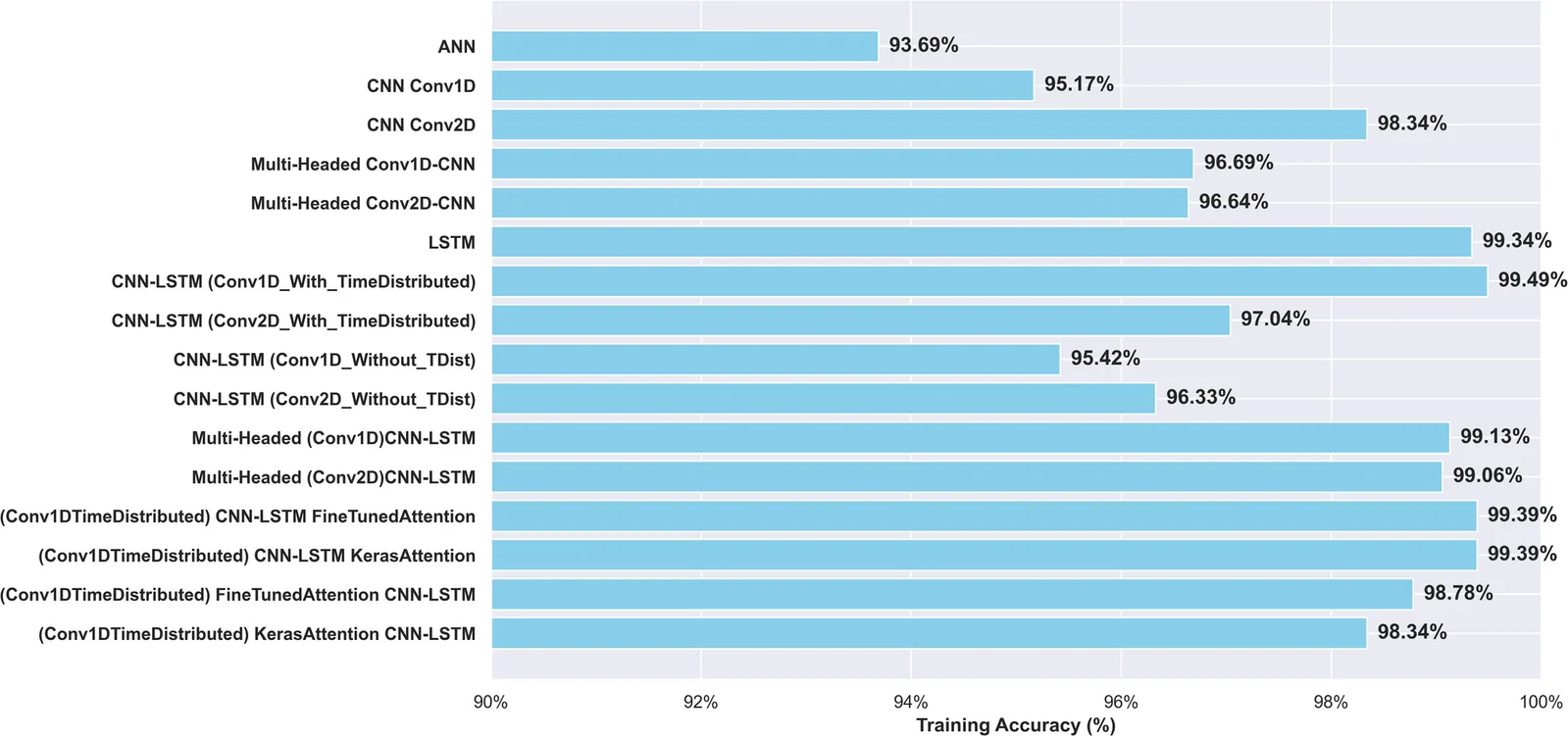
Training accuracy comparison on the SDALLE dataset.

The performance on SDALLE is reported on the same randomised window-level evaluation protocol as for the ENABL3S benchmark in our previous work^20^. This allows for a descriptive comparison under like processing choices, but does not provide an independent estimate of performance with unseen participants. The training accuracies of the tested architectures are divided into three groups and are shown in Fig. 16:

(i) Baseline architectures (ANN, CNN, LSTM).
(ii) Hybrid CNN–LSTM models.
(iii) Attention-enhanced CNN–LSTM variants.

Baseline models, including ANN and standalone CNN variants, achieved training accuracies in the range of approximately 93–98%. The LSTM architecture demonstrated improved training performance, reaching approximately 99%. Hybrid CNN–LSTM configurations and attention-enhanced variants consistently achieved training accuracies exceeding 98–99%, indicating strong capacity to fit the training data.

**Fig. 17:**
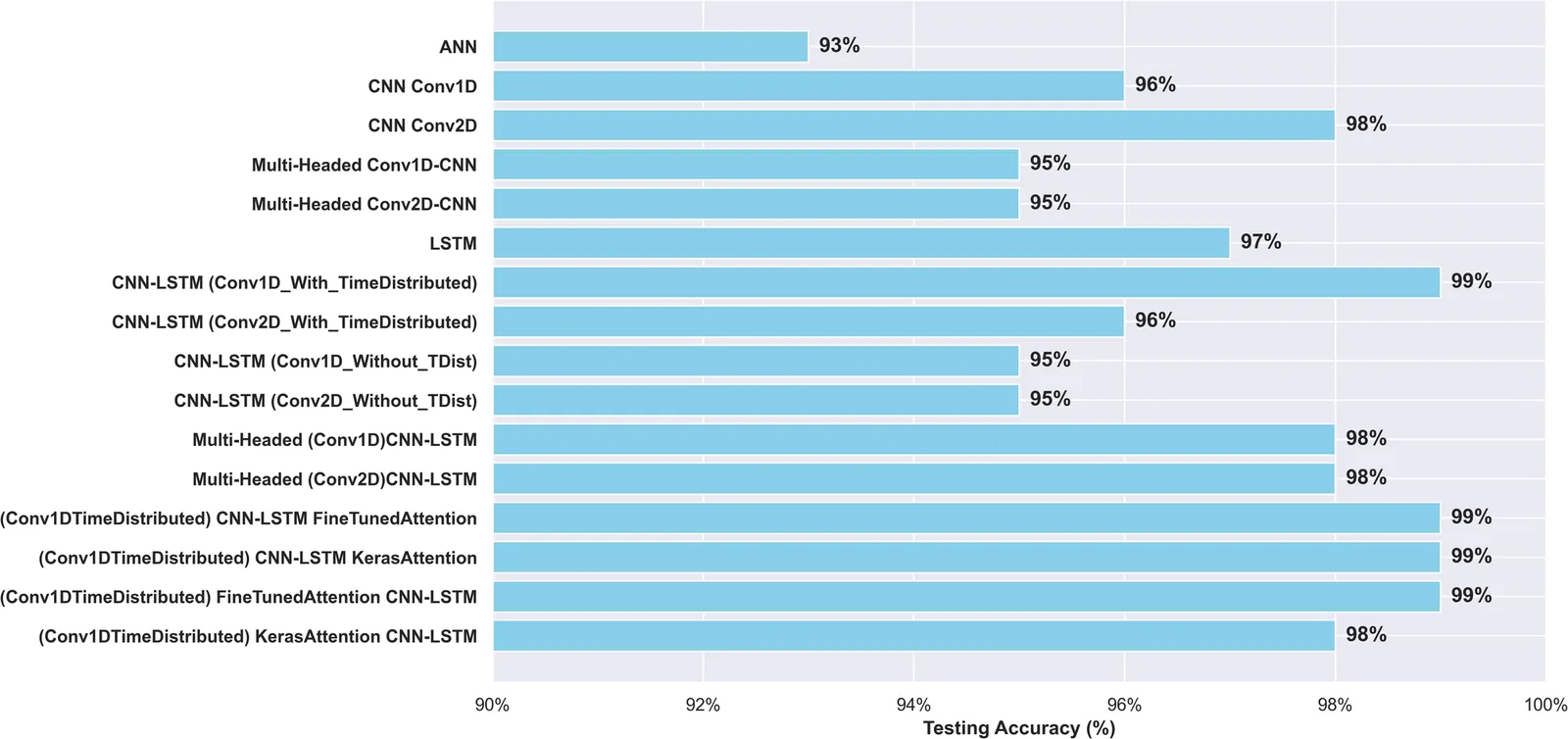
Testing accuracy comparison on the SDALLE dataset.

**Fig. 18:**
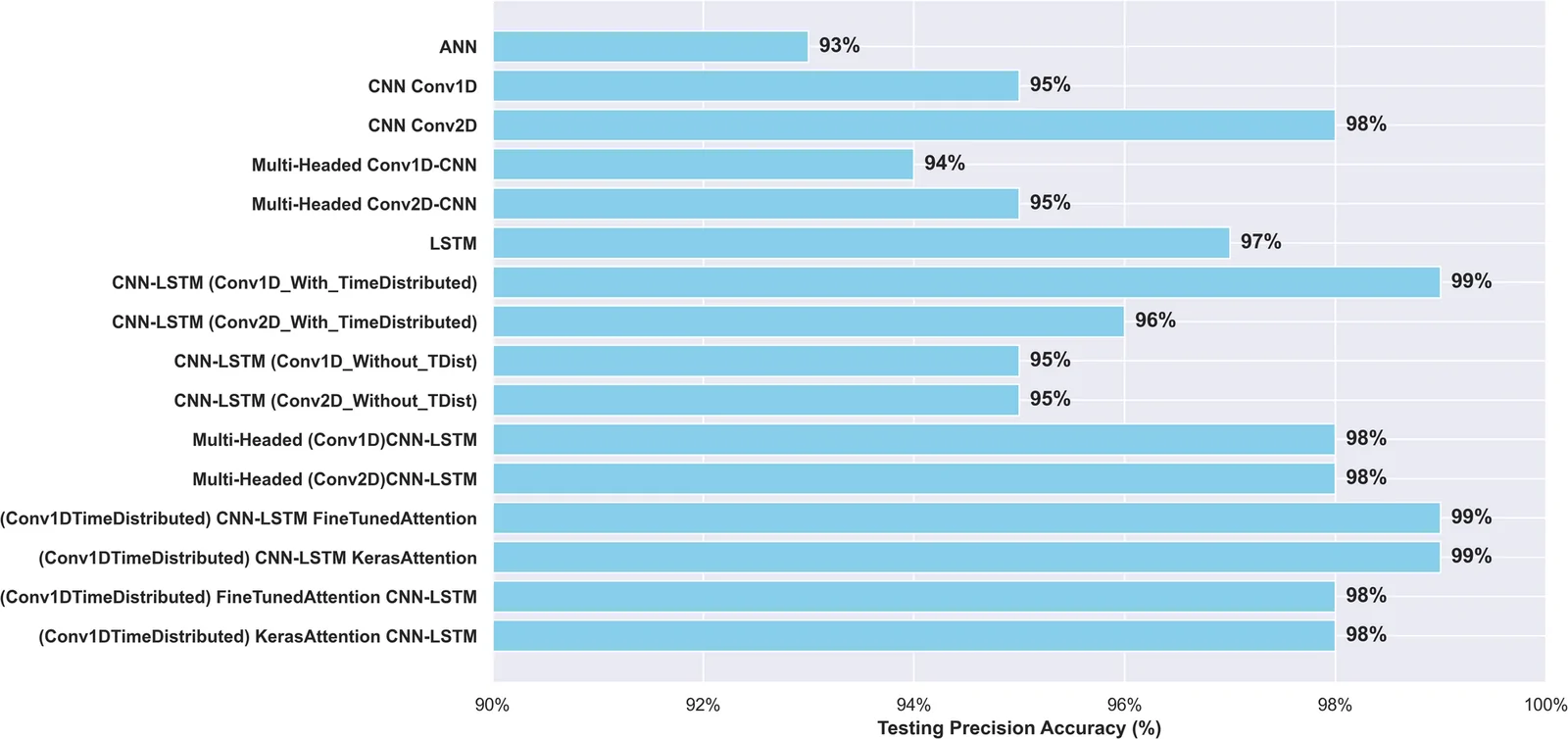
Testing precision comparison on the SDALLE dataset.

**Fig. 19:**
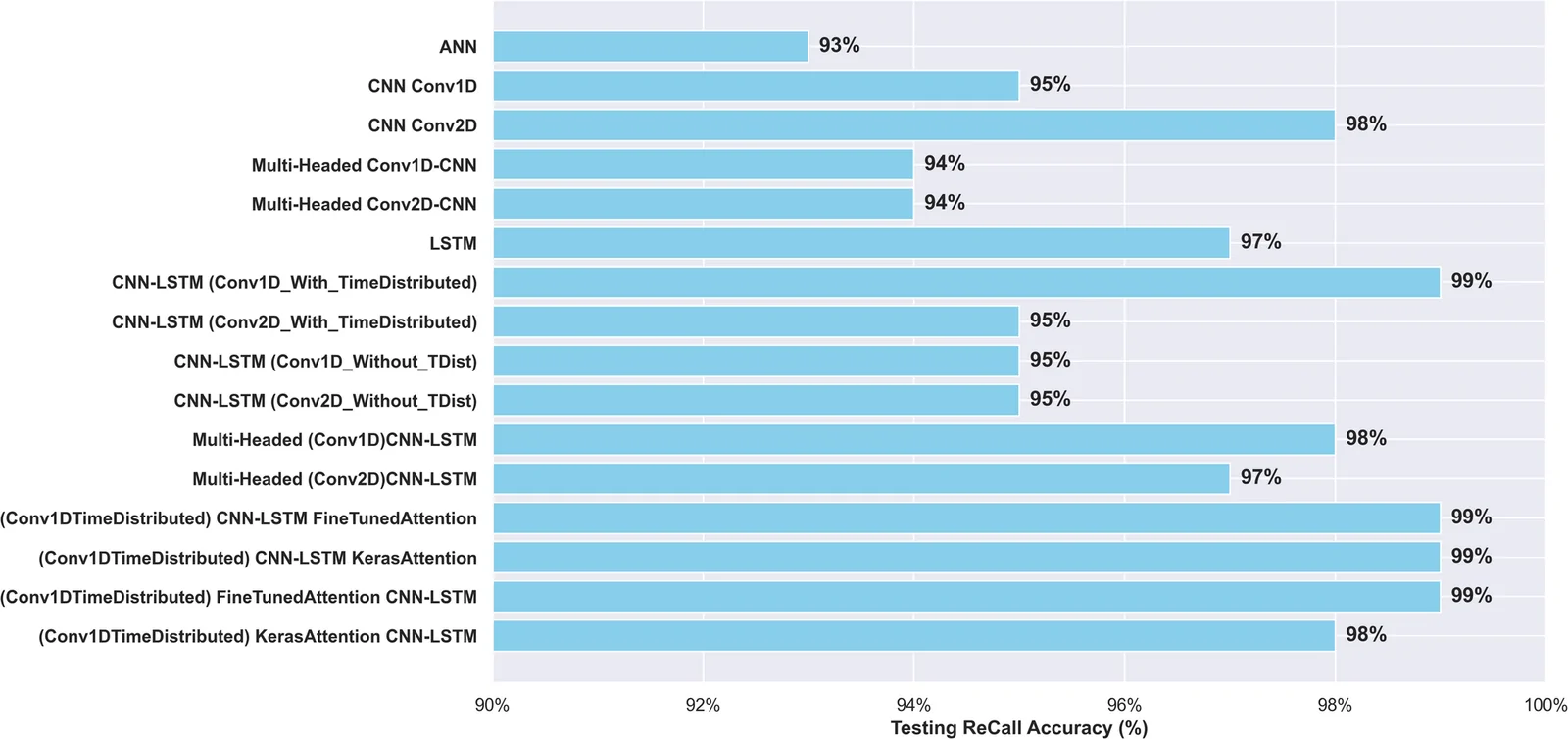
Testing recall comparison on the SDALLE dataset.

**Fig. 20:**
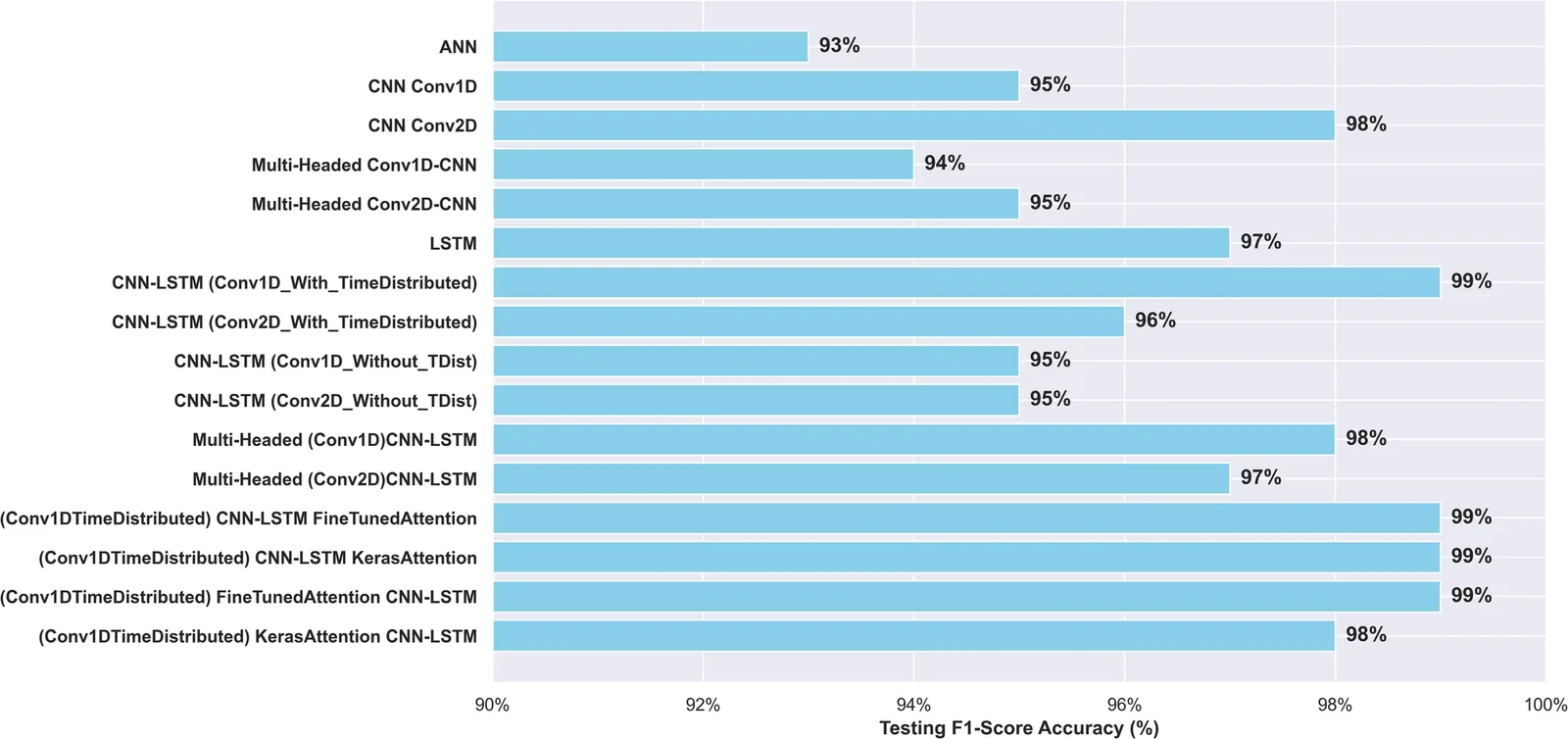
Testing F1-score comparison on the SDALLE dataset.

The training accuracy indicates the ability of the model to fit the data. The accuracy, precision, recall, and F 1 score on the subject-dependent randomised window-level test subset are summarised in Table 8; Figs. 17, 18, 19 and 20. These measurements are conditional on the partitioning procedure and not participant-independent generalisation estimates.

In total, we have evaluated 16 architecture variants. To make the tabular presentation more concise, we report in Table 8 ten representative configurations covering the baseline, convolutional, recurrent, hybrid and attention-based model families. Figures 17, 18, 19 and 20 display the complete model-level results for the 16 variants tested. The tables presented here are only for illustration and do not affect the models included in the aggregate analysis.

**Table 8:** Model-level performance of 10 representative architectures selected from the 16 evaluated variants on the SDALLE dataset (subject-dependent randomized window-level evaluation).

Model	Accuracy (%)	Precision (%)	Recall (%)	F1-score (%)
ANN	93.1	93.0	92.8	92.9
CNN Conv1D	95.6	95.4	95.2	95.3
CNN Conv2D	97.2	97.0	96.9	96.9
Multi-Head CNN	97.8	97.6	97.5	97.5
LSTM	97.1	97.0	96.8	96.9
CNN–LSTM (Sequential)	98.4	98.3	98.2	98.2
CNN–LSTM (TimeDistributed)	98.7	98.6	98.5	98.5
Multi-Head CNN–LSTM	98.9	98.8	98.7	98.7
CNN–LSTM + Keras Attention	99.0	98.9	98.9	98.9
CNN–LSTM + Fine-Tuned Attention	99.2	99.1	99.0	99.0

Under the randomized window-level protocol, attention-enhanced CNN–LSTM models recorded the highest measured values, with accuracy, precision, recall, and F1-score approaching 99%. The hybrid CNN-LSTM models without attention also yielded high values over the same test subset. These numbers may be optimistic as the training data may contain related windows from participants and trials that are in testing, and should not be interpreted as performance on unseen participants.

Within the same subject-dependent test subset, the ANN baseline recorded approximately 93% across the evaluation metrics, standalone CNN models ranged between approximately 95% and 98%, and the LSTM-only architecture recorded approximately 97% accuracy. These inter-model differences describe behaviour under the implemented split only; overlapping information may affect architectures differently.

The attention-based hybrid models balanced precision and recall in the randomised window level test subset. This pattern is descriptive of the current protocol and does not show robustness or predictive consistency across unseen participants. We also studied computational characteristics such as training time over 30 epochs and inference time which we discuss in the following subsection.

### Computational analysis

**Fig. 21:**
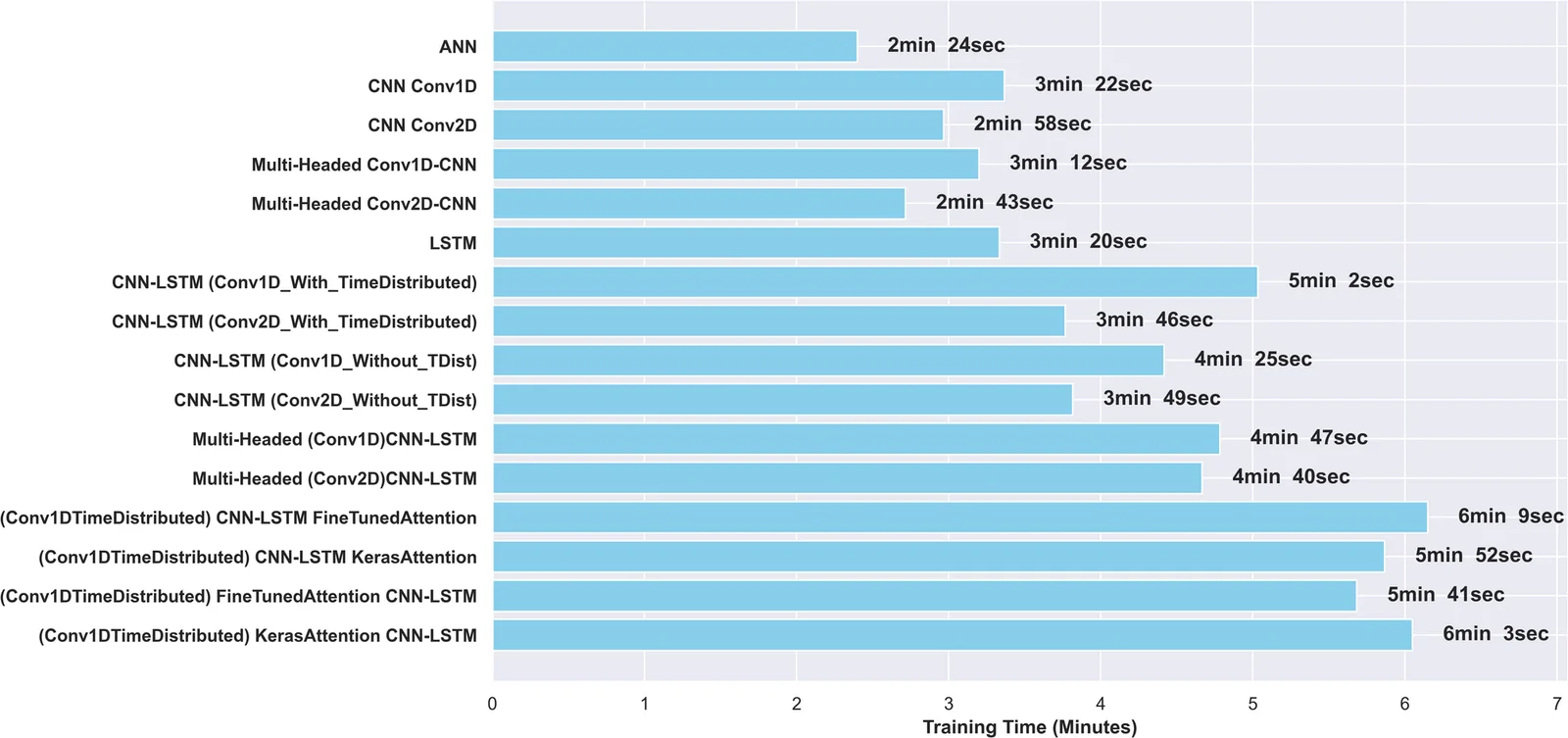
Training time comparison on the SDALLE dataset.

**Fig. 22:**
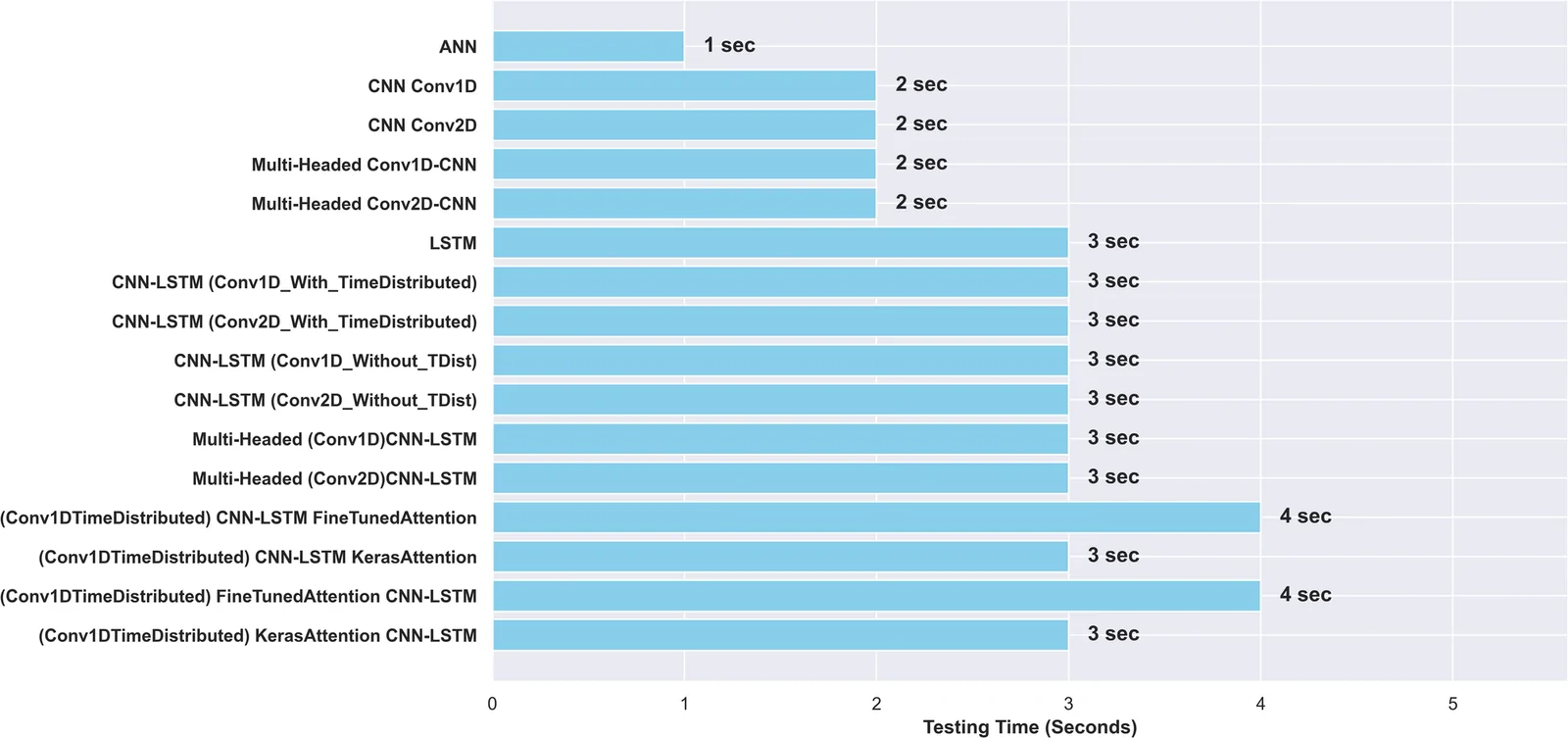
Testing time comparison on the SDALLE dataset.

Figures 21 and 22 show the training and inference time of each architecture to run 30 epochs on the SDALLE dataset. Table 9. Approximate number of trainable parameters and computational characteristics of deep learning architectures evaluated on SDALLE dataset in the unified experimental framework.

**Table 9:** Computational complexity and execution time of evaluated architectures on the SDALLE Dataset.

Model	Trainable Parameters (Approx.)	Training Time (30 Epochs)	Testing Time
ANN	~ 0.45 M	Lowest	Very Low
CNN Conv1D	~ 0.85 M	Low	Low
CNN Conv2D	~ 1.10 M	Low–Moderate	Low
Multi-Head CNN	~ 1.45 M	Moderate	Low
LSTM	~ 3.80 M	Moderate–High	Moderate
CNN–LSTM (Sequential)	~ 4.20 M	High	Moderate
CNN–LSTM (TimeDistributed)	~ 4.50 M	High	Moderate
Multi-Head CNN–LSTM	~ 5.10 M	High	Moderate
CNN–LSTM + Keras Attention	~ 5.40 M	Very High	Moderate
CNN–LSTM + Fine-Tuned Attention	~ 5.70 M	Highest	Moderate

The computational cost increases with the complexity of the architecture as expected. The Artificial Neural Network baseline had the shortest training time, reflecting its fully connected structure and the lack of sequential dependencies. Standalone Convolutional Neural Network models (Conv1D and Conv2D) also demonstrated relatively efficient training, benefiting from parallelizable convolutional operations and moderate parameter counts. The Long Short-Term Memory model demanded longer training time than feedforward and convolutional baselines. Recurrent computations are by nature sequential across time steps, and therefore limit the efficiency of parallelisation. However, the chosen stacked configuration in this work was computationally feasible and imposed a moderate training load.

The hybrid CNN and LSTM architectures incurred some computational burden in combining the convolutional feature extraction with recurrent temporal modelling. Configurations using Time Distributed convolutional operations and multi-headed branches took even longer to train. CNN-LSTM with attention variants required the most training time. The attention mechanism adds learnable projections and temporal weighting operations. Inference times measured on the study workstation remained relatively low, but these measurements characterise the implemented hardware and window-level pipeline and do not provide validation in a deployed wearable environment.

Overall, the measurements characterise a protocol-specific trade-off between computational cost and observed window-level test performance. Under the current split, attention-augmented hybrid architectures achieved higher test metrics and longer training times, while simpler models needed less computation. Note that this comparison does not represent a definitive ranking for participant-independent use since independence from test set was not ensured.

### Confusion matrix analysis

**Fig. 23:**
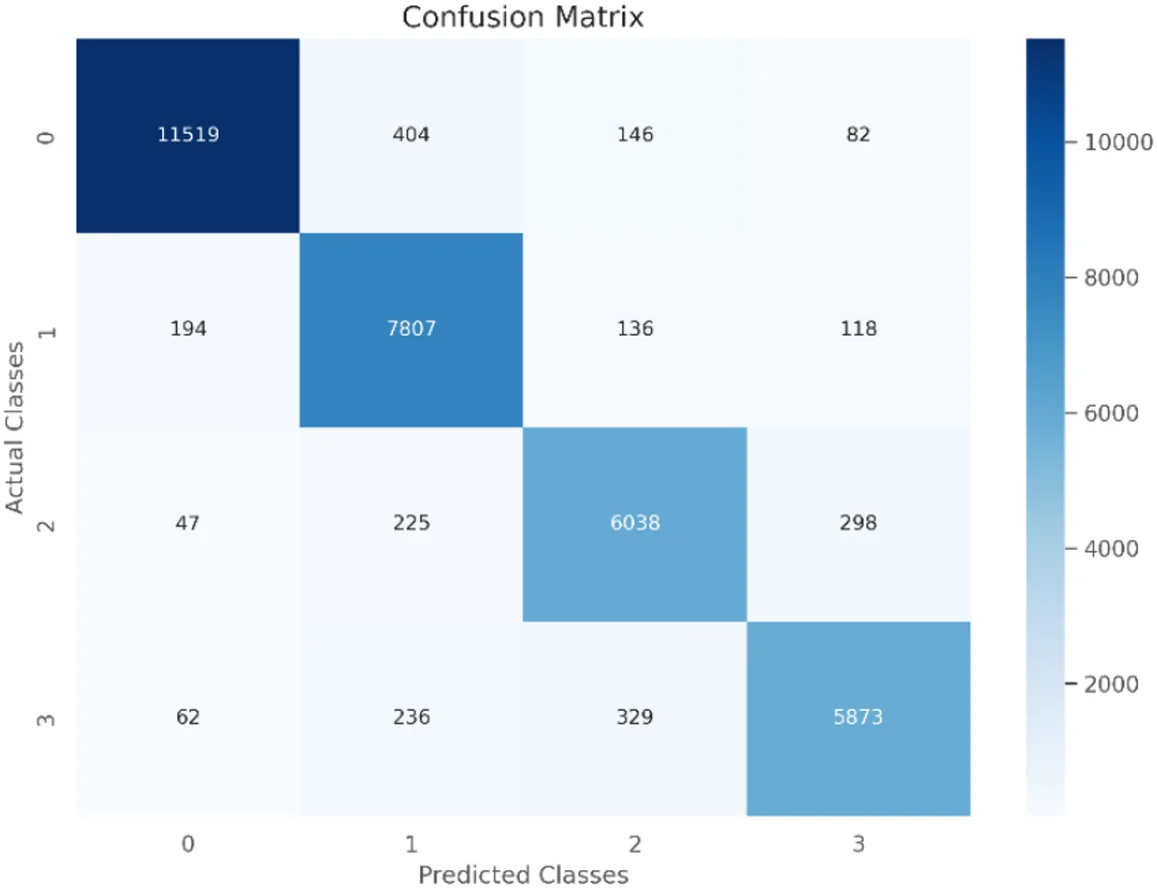
Confusion matrix – ANN – SDALLE dataset.

**Fig. 24:**
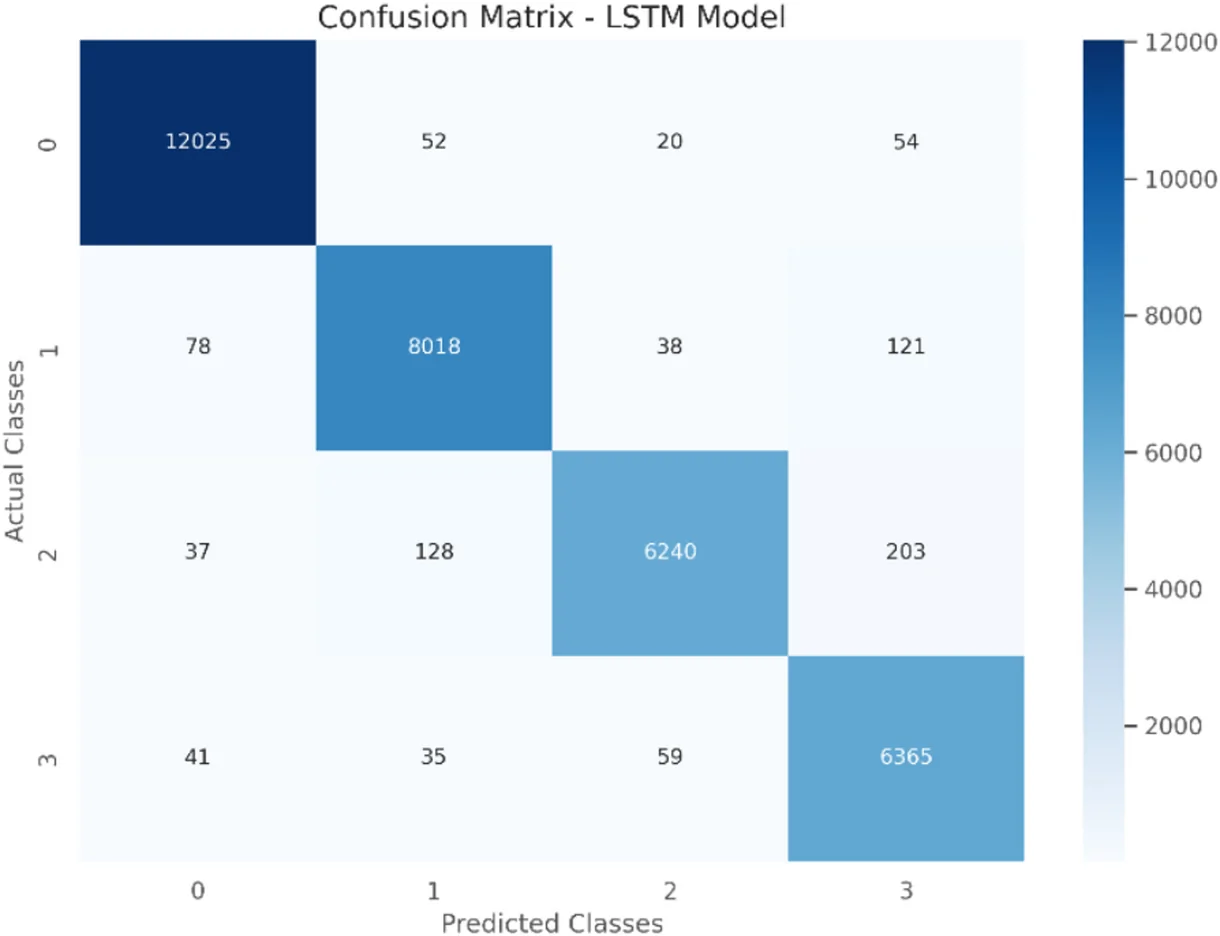
Confusion matrix – LSTM – SDALLE dataset.

**Fig. 25:**
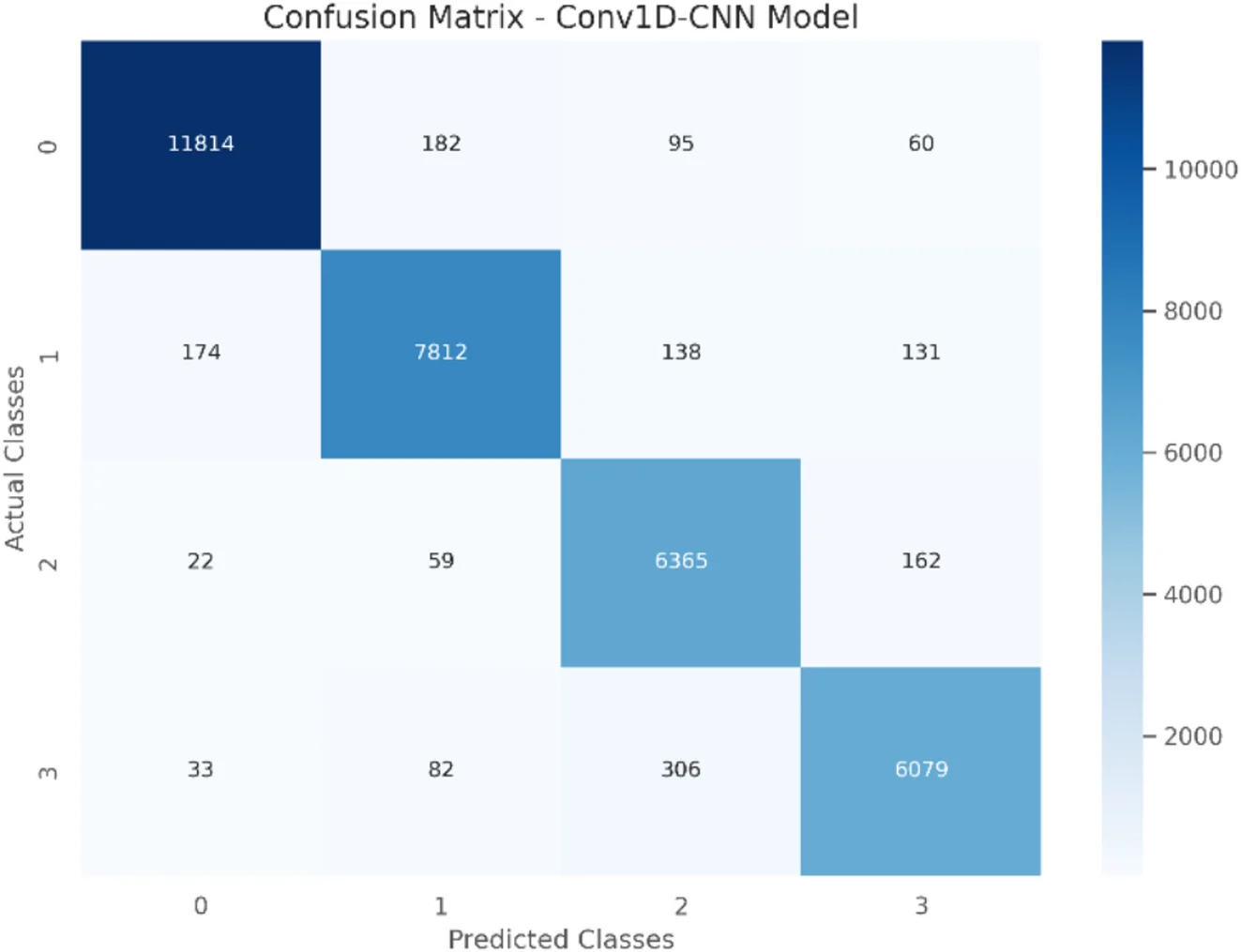
Confusion matrix – CNN Conv1D – SDALLE dataset.

**Fig. 26:**
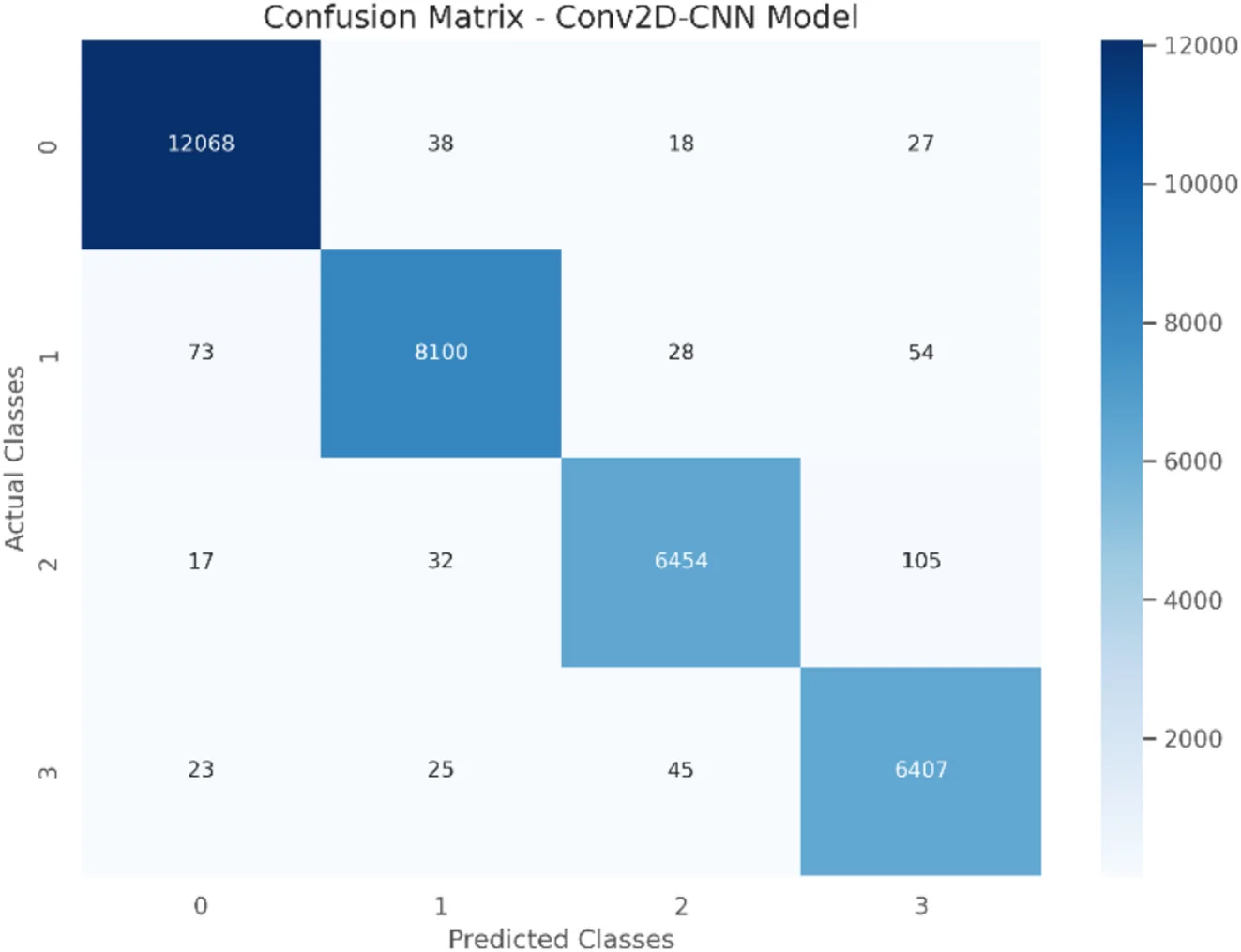
Confusion matrix – CNN Conv2D – SDALLE dataset.

**Fig. 27:**
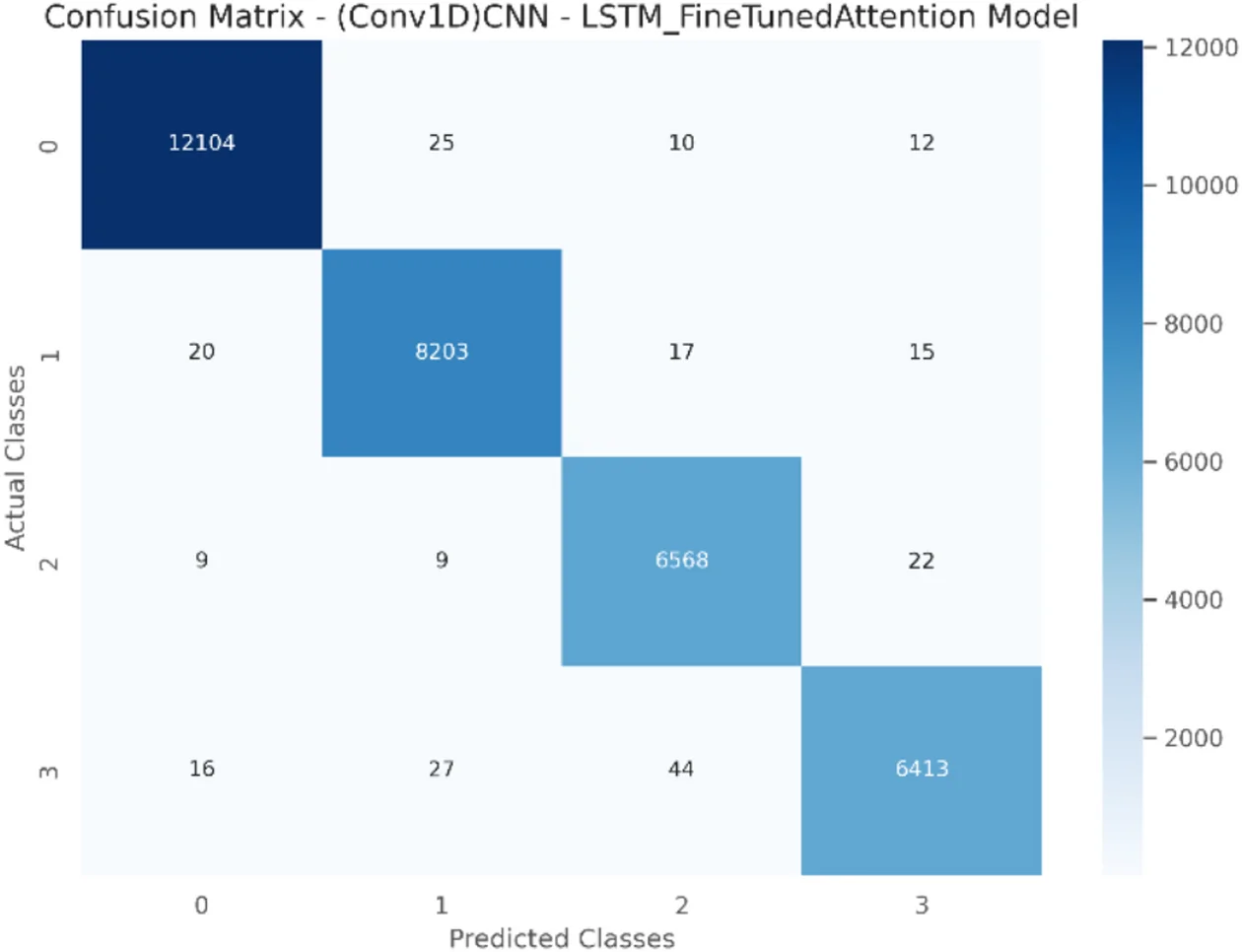
Confusion matrix – CNN-LSTM FineTunedAttention – SDALLE dataset.

**Fig. 28:**
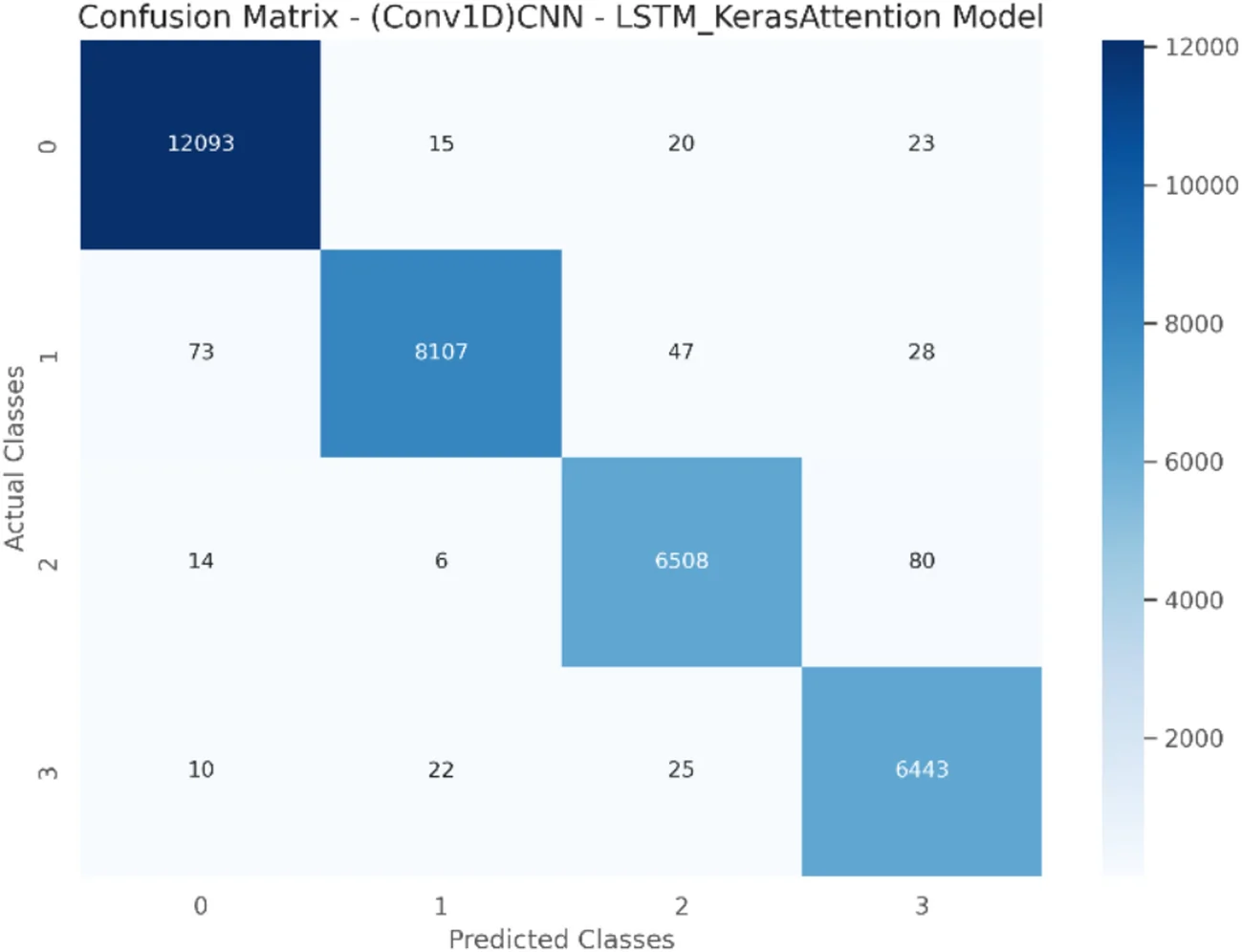
Confusion matrix – CNN-LSTM KerasAttention – SDALLE dataset.

**Fig. 29:**
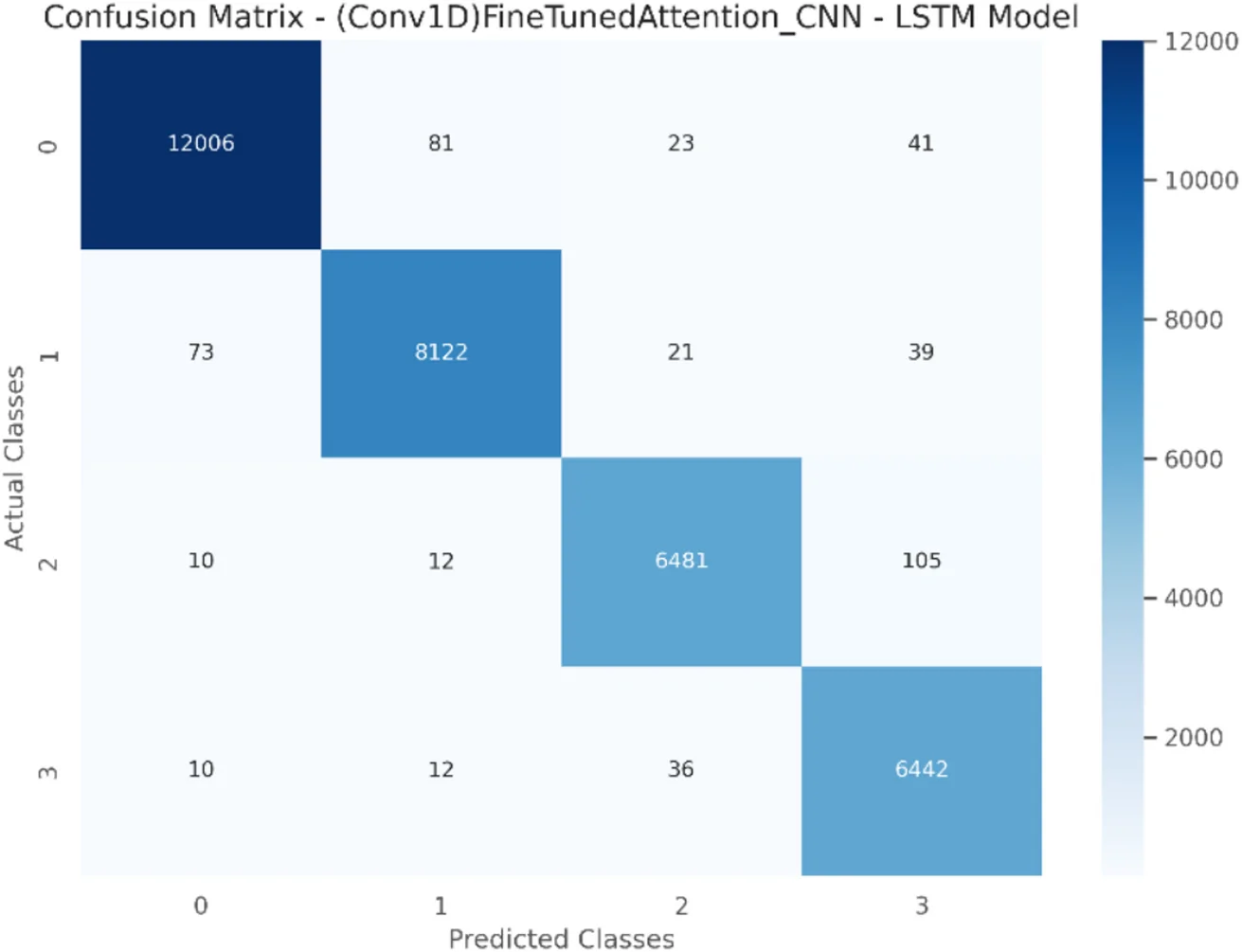
Confusion matrix – FineTuned Attention CNN-LSTM – SDALLE dataset.

**Fig. 30:**
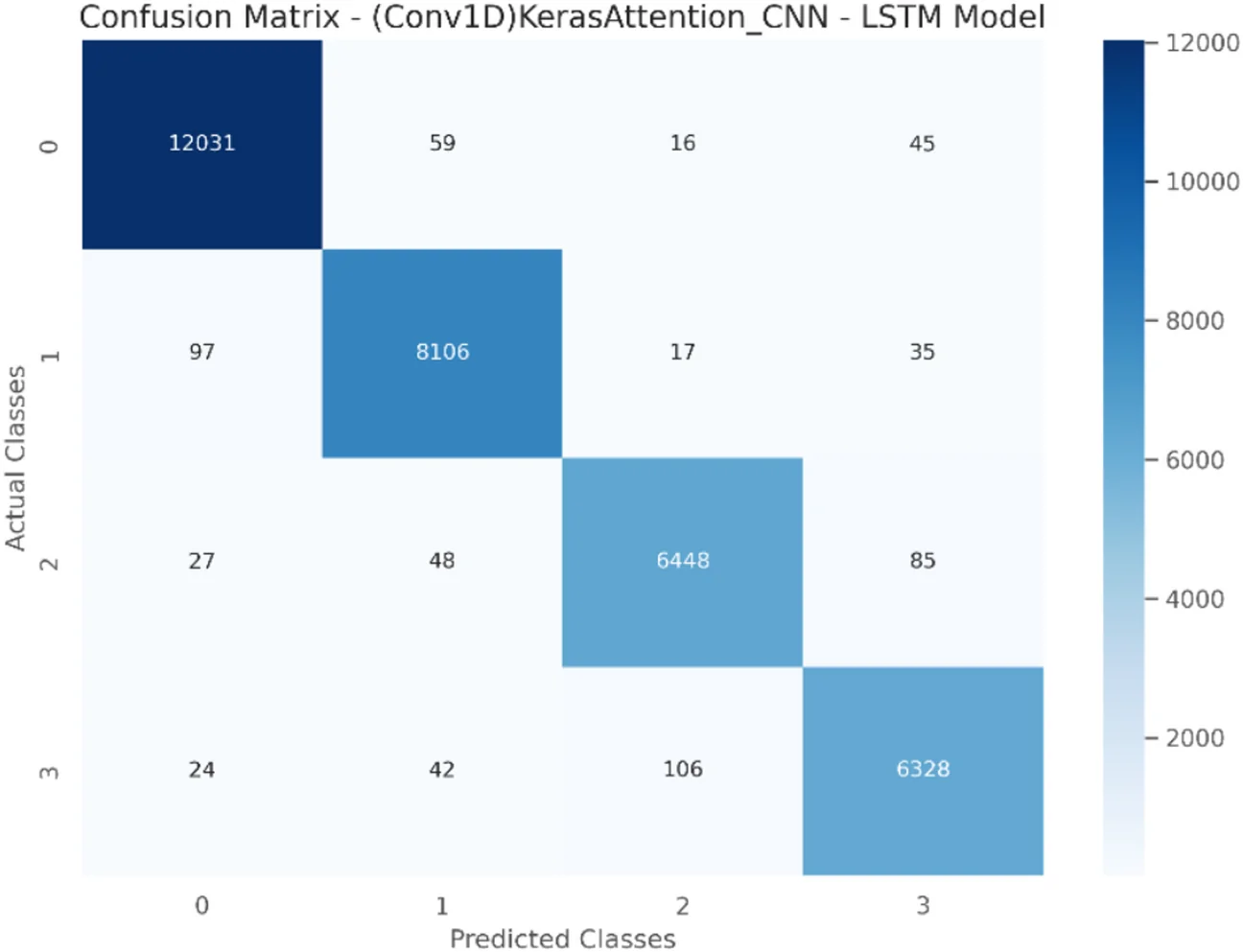
Confusion matrix – Keras Attention CNN-LSTM – SDALLE dataset.

Figures 23, 24, 25, 26, 27, 28, 29 and 30 show confusion matrices for the evaluated architectures on the SDALLE randomised window-level test subset. Class indices are Walking (0), Jogging (1), Stairs Up (2), Stairs Down (3). Strong diagonal patterns were obtained, with 12,093 correct predictions of Walking, 8,107 of Jogging, 6,508 of Stairs Up and 6,443 of Stairs Down. However, as windows from participants and trials seen in training can also appear in testing, and adjacent windows can share raw samples, these matrices do not reflect independent classification of unseen participants, and their diagonal dominance may be optimistic.

The off-diagonal entries in the test subset implemented were mainly between Walking and Jogging and between Stairs Up and Stairs Down. For the baseline models, there were more such entries than in the hybrid and attention enhanced variants. This pattern of description is consistent with differences in the measured metrics at the window level, but the role of architectural design cannot be dissociated from the possibility that the models utilised overlapping or participant specific information. Thus, the matrices do not give better discrimination for unknown subjects.

In general, the confusion matrices agree with the previously reported subject-dependent window level metrics. They should not be taken as evidence of cross-subject robustness. The following subsection presents the results of the exploratory GAN augmentation analysis with the same partitioning constraint.

### Exploratory impact of GAN-based data augmentation under subject-dependent evaluation

**Fig. 31:**
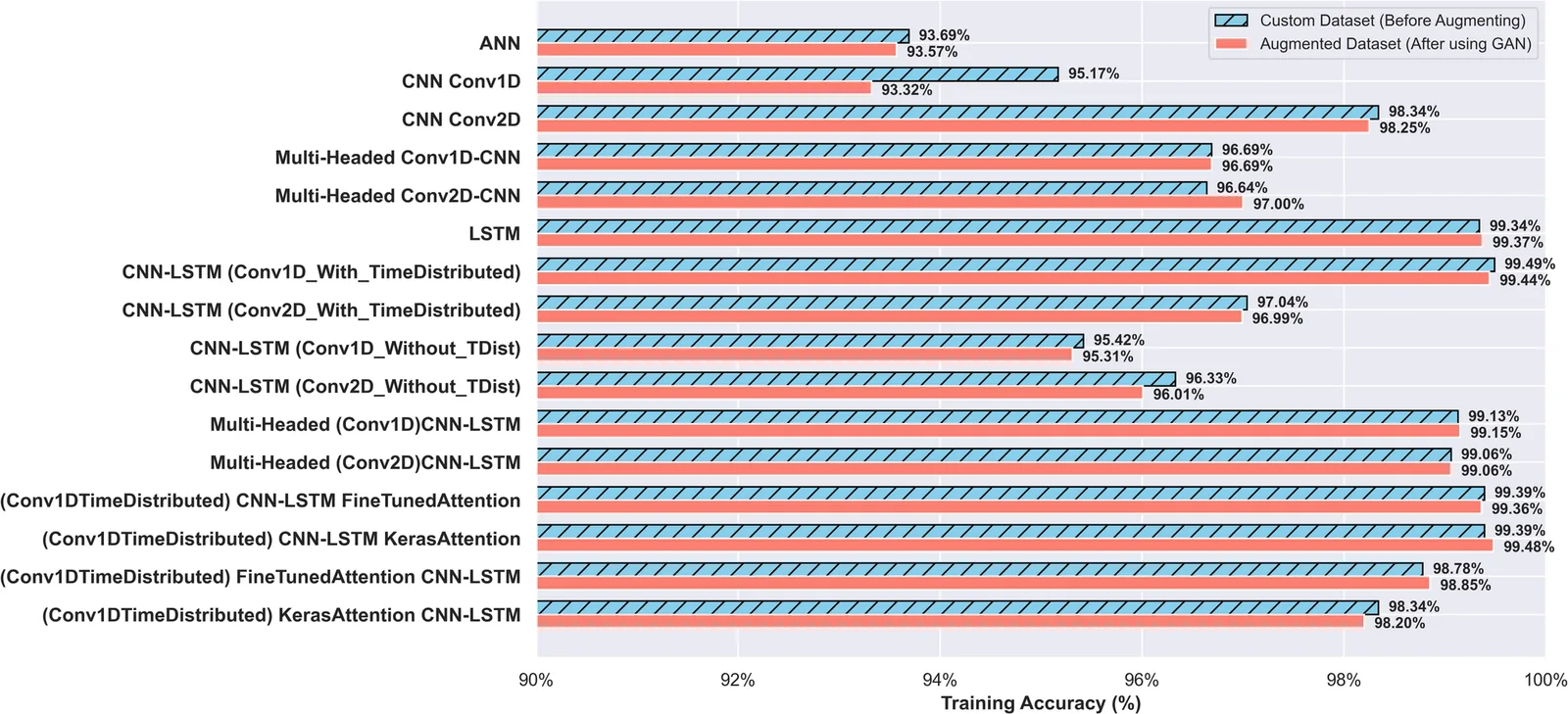
Training accuracy comparison before and after GAN-based augmentation.

**Fig. 32:**
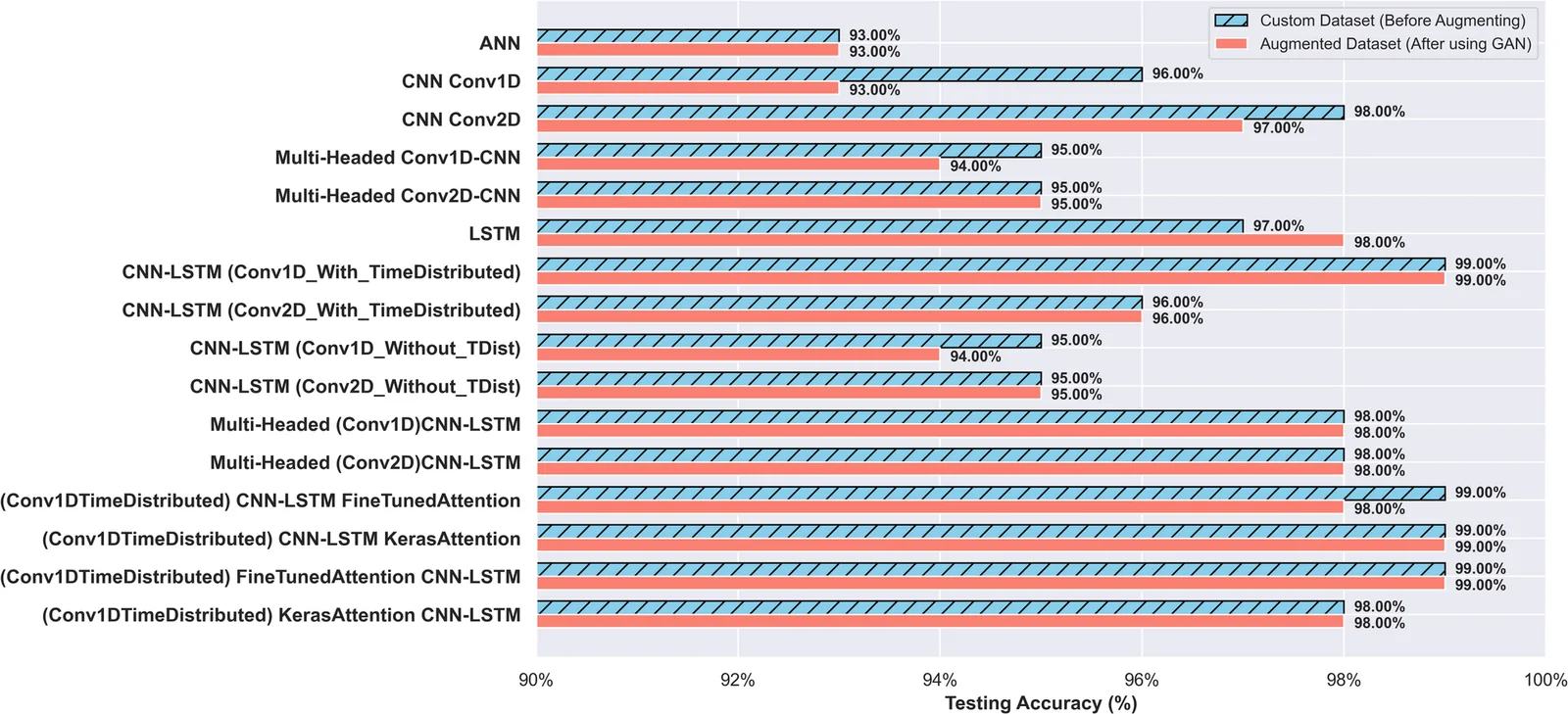
Testing accuracy comparison before and after GAN-based augmentation.

This subsection investigates the behaviour of the model before and after GAN-based augmentation on SDALLE, utilising the F1-score, precision, recall, and accuracy. The same randomised 70:30 window-level partitioning procedure was used to evaluate both conditions. However, the original and augmented datasets were separately partitioned as the generated data from GAN was added before partitioning and no identical testing subsets were found in the datasets. As above, GAN generation and preprocessing were done before the final split, not within participant-independent training folds. Thus, the analysis is limited to exploratory class-balancing behaviour in the current subject-dependent protocol and does not demonstrate improved generalisation, robustness, or synthetic-signal validity. In future work, a full validation of the generated signals will be performed, on the task level and distributionally. Figures 31 and 32 shows the comparison of training and testing accuracies before and after the GAN based augmentation.

Training results suggest that the augmentation did not significantly alter the fitting accuracy of the higher-capacity architectures which stayed close to 99% under the applied procedure. The ANN and Conv1D CNN were slightly different. These training values do not determine whether augmentation improves generalisation, and cannot be used to rule out inflation associated with the non-independent partition.

**Figs. 33:**
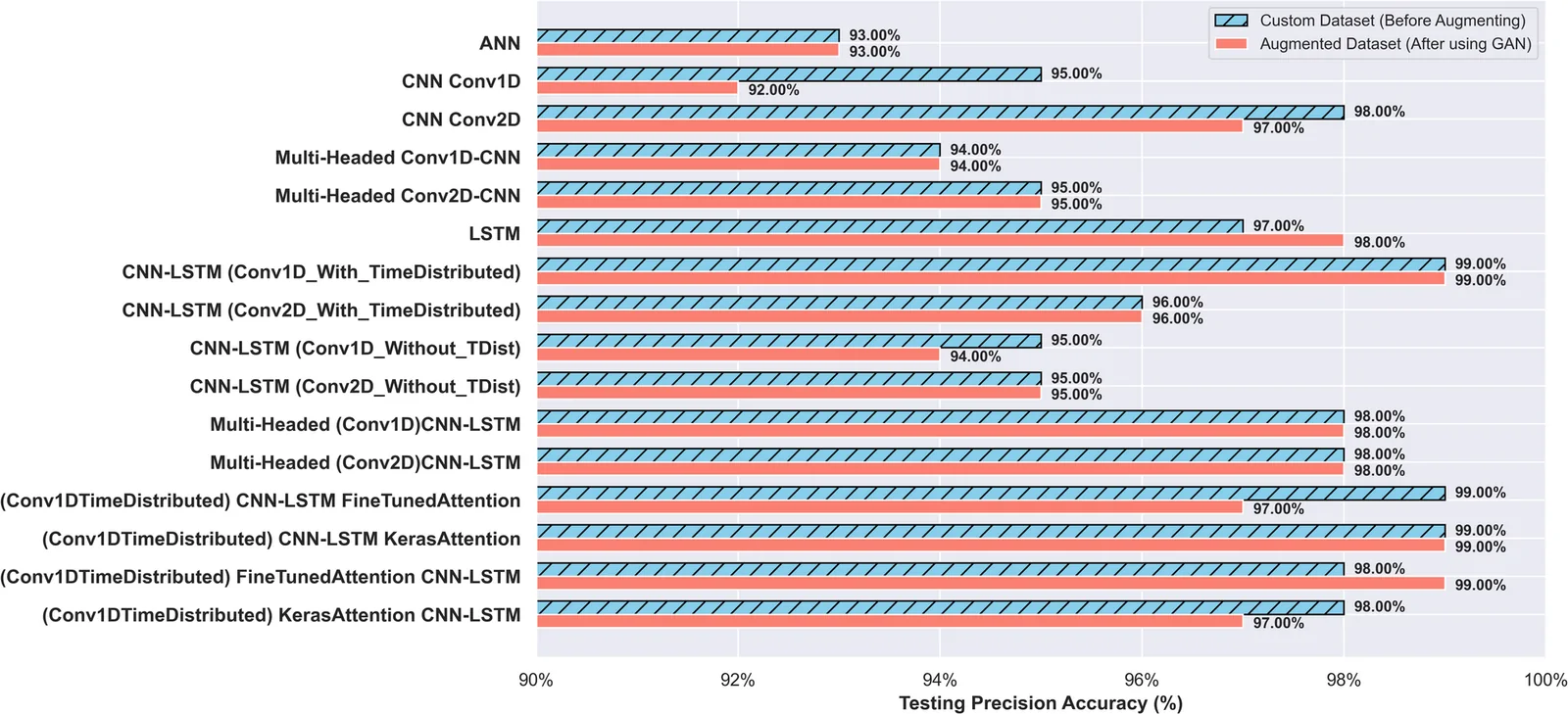
Testing Precision comparison before and after augmentation.

**Fig. 34:**
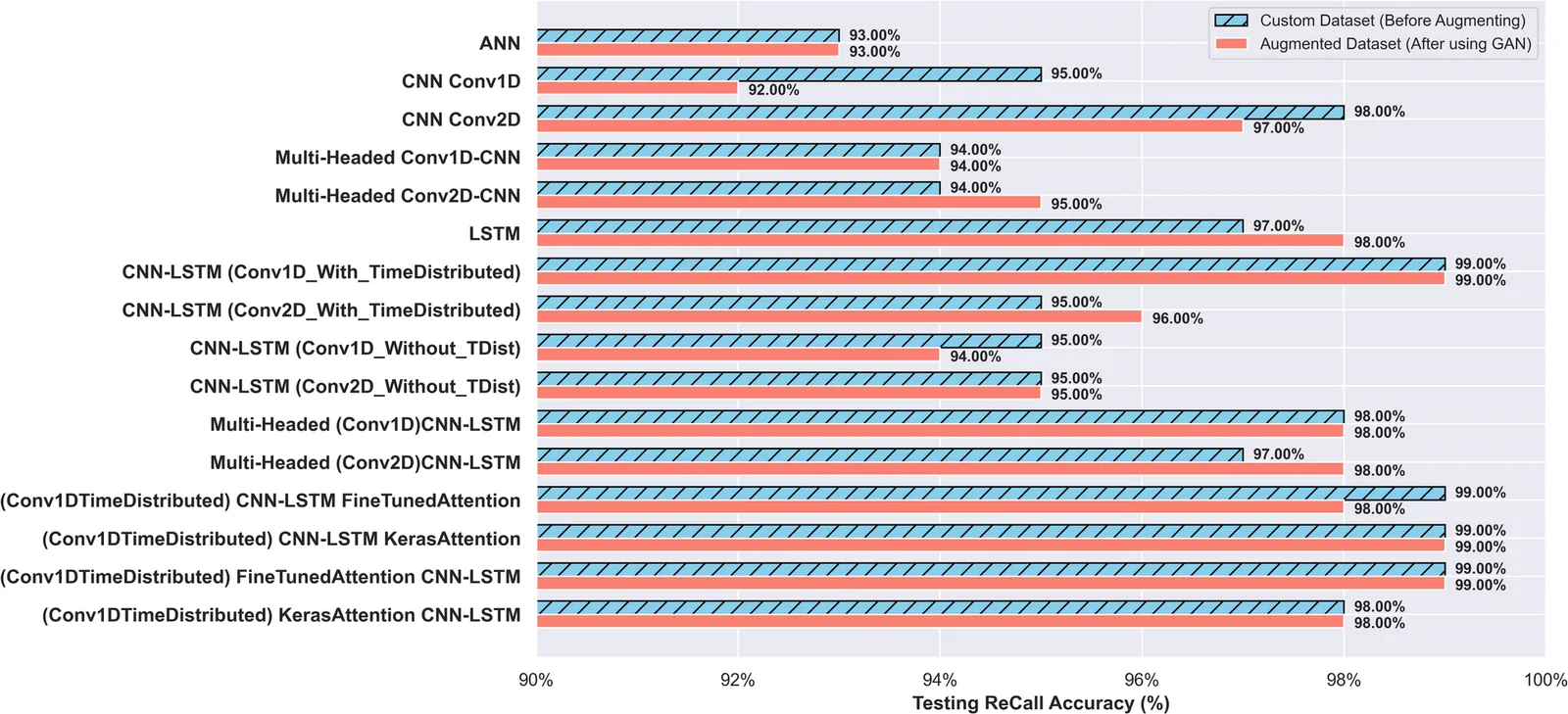
Testing Recall comparison before and after augmentation.

**Fig. 35:**
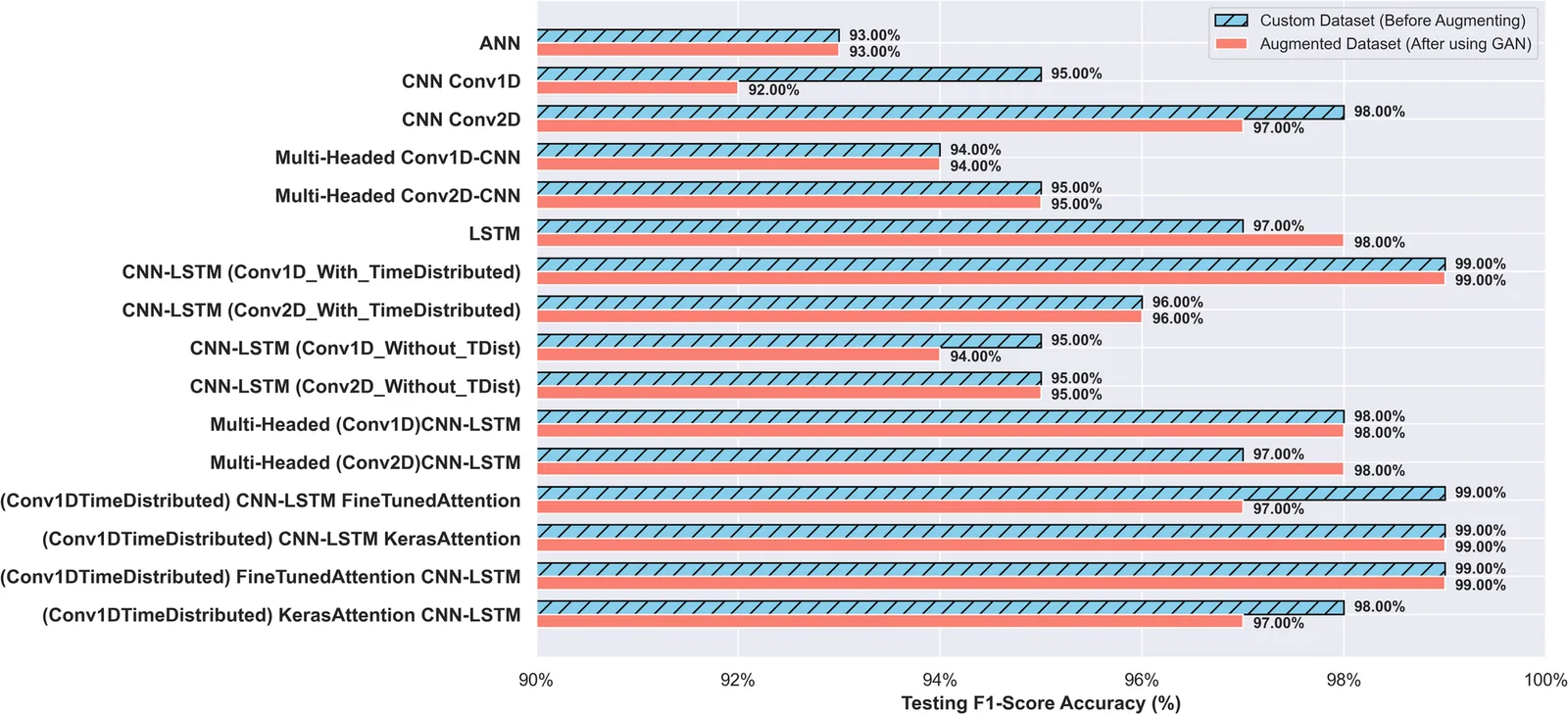
Testing F1-Score comparison before and after augmentation.

Figures 33, 34, 35 summarise the testing results of precision, recall and F1-score before and after augmentation on the same subject-dependent randomised window-level test protocol.

Table 10 shows average testing performance of all 16 model variants shown individually in Figs. 32, 33, 34, 35. each model produced one result for each condition, and both were weighted equally in the calculation. Table 10 was thus calculated on the full set of 16 models, rather than the ten representative configurations of Table 8.

**Table 10:** Average testing performance of all 16 evaluated model variants before and after GAN-based augmentation on the SDALLE dataset (subject-dependent randomized window-level evaluation).

Metric	Before GAN (%)	After GAN (%)
Testing Accuracy	96.9	96.5
Precision	96.7	96.3
Recall	96.6	96.4
F1-score	96.7	96.3

For the 16 models compared, the average testing accuracy was 96.9% before, and 96.5% after augmentation, a descriptive change of -0.4% points. Average precision decreased from 96.7% to 96.3%, recall from 96.6% to 96.4% and F1-score from 96.7% to 96.3% CNN-LSTM and attention-based variants with larger capacity generally stayed in the 97–99% range, while some simpler architectures exhibited more variation.

Such modifications are but descriptive of. Performance was not analysed across classes, variation between repeated runs, confidence intervals, effect sizes or inferential statistical tests. Thus, the presented results do not demonstrate that GAN augmentation has reduced majority-class bias, improved prediction stability or improved robustness. The direct documented effect of the augmentation is the increasing of 54,000 synthetic rows to the three minority activities with the walking class proportion changing from 36.3% to 32.4%.

## Discussion

A central contribution of this study is the SDALLE resource and the systematic evaluation of established models under a harmonized multimodal EMG–IMU pipeline across SDALLE and ENABL3S, rather than the introduction of new neural architectures. The attention enhanced CNN-LSTM variants performed best under randomised window-level protocol on both datasets whereas ANN and standalone CNN variants performed lower. This ordering is descriptive and protocol-specific as the testing sets were not independent at the participant or trial level.

The repeated appearance of hybrid and attention-based models at the top of the measured rankings may reflect the complementary roles of convolutional, recurrent and attention operations in multimodal time-series modelling. However, the current design does not exclude that this pattern is due to a portable inductive bias rather than participant-specific or overlapping-window information. You need outside confirmation of that reading, other than the subjects.

The errors in the present test subsets were mainly between walking and jogging, and between stair ascent and descent. Hybrid or attention-based models had less off-diagonal entries. These patterns are consistent with better modelling of temporal structure, but they are still hypotheses, not evidence of an architecture-specific benefit for unseen participants, since the confusion matrices were computed from correlated, participant-mixed windows.

Model rankings observed separately on SDALLE and ENABL3S were broadly similar with harmonised choices of processing. This descriptive recurrence does not generalise across datasets or subjects as neither evaluation was performed on participant-independent partitions, and correlated windows may affect the different architectures differently. Thus, the rankings should be taken as specific to the subject-specific protocols implemented and not as evidence that the models avoided dataset-specific or participant-specific information.

The computational measurements observed provide context for model selection in this experiment. The hybrid and attention enhanced models took more time for training because of the combined convolutional, recurrent and attention operations whereas the ANN and standalone CNN models took less time. The study workstation still showed low inference time. These measurements do not confirm real-time performance on wearable hardware and the apparent accuracy advantage has to be re-evaluated under participant-independent validation before deployment-oriented conclusions are drawn.

One possible signal-level explanation for the observed within-protocol behaviour is that attention applies explicit temporal weighting to activity-discriminative portions of a window, while CNN–LSTM models encode temporal dependencies without this weighting. Locomotor phases such as foot contact, propulsion and elevation transition may contain useful information. However, the balanced precision-recall profiles for the present test subset are not a robustness test, this proposed mechanism needs to be tested in a participant-independent data.

The GAN augmentation analysis is an experimental one. Aggregate accuracy was not much different, high-capacity models were still high and simple models had more variability. Because augmentation was performed before the participant-mixed randomized split, the analysis cannot determine whether synthetic data improve generalization or class balance for unseen participants. It only logs the behaviour of downstream classifiers in the implemented window-level protocol.

Another limitation is the quality and location of the GAN samples within the validation pipeline. The study did not compare different generative models or perform extensive distributional validation of synthetic signals. Further, the GAN fitting was not repeated over participant independent training folds. Therefore, augmentation is only considered as an exploratory analysis for balancing classes. Future work should train the GAN individually on each training fold, excluding some participants for testing on their real data and assessing the synthetic quality with distributional and task-level protocols such as Maximum Mean Discrepancy and Train-on-Synthetic-Test-on-Real.

Methodological considerations are relevant to the interpretation of these results. Consistent optimisation settings reduce procedural variation across models but do not address the lack of test-set independence. In addition, the electromyography was downsampled to the inertial sampling rate by cubic interpolation to allow EMG-IMU synchronisation. Visual inspection indicated that the envelopes of activation and timing structure were preserved but the lower temporal resolution attenuated higher frequency EMG content. Thus, the results are valid for the specified synchronised representation and subject dependent evaluation protocol.

Several limitations must be taken into account in interpreting these findings. The SDALLE dataset is a relatively small and demographically homogenous cohort of healthy male participants limiting demographic generalisability. Furthermore, data acquisition was performed under controlled laboratory conditions using a simplified three-step staircase setup that was designed to ensure safe and repeatable multimodal recordings. This set-up improved consistency of acquisition but may not adequately capture the variability and complexity of natural daily-life stair negotiation and unconstrained locomotor behaviour. Moreover, the study specifically focused on four lower-limb locomotor activities that are relevant for rehabilitation and wearable assistive applications; hence, the findings might not directly generalise to broader activity recognition scenarios.

Another practical limitation is the feasibility of the multi-sensor wearable set-up. The DELSYS Trigno sensors used in this study are compact and wireless, however the experimental protocol involved the use of multiple sensors attached bilaterally to the lower limbs with adhesive interfaces and elastic fixation straps. This configuration was suitable for controlled laboratory data acquisition and allowed stable multimodal EMG-IMU recording during short experimental sessions. However, formal assessment of user comfort, donning/doffing time, long-duration attachment stability, and participant acceptability through a structured questionnaire was not included in the study. Thus, the current implementation should be viewed as a research-quality multimodal acquisition setup rather than as a final deployable wearable system. Future work should include investigating reduced-sensor configurations, sensor-placement optimisation, and formal usability evaluation for extended clinical or daily-life use.

The main limitation in methodology is the train/test split. The feature standardisation was fitted on the complete merged dataset, and signals were segmented with 75%-overlapping windows and randomly split 70:30 at the window level. No participant level or trial level grouping was done. For SDALLE, 12/16 samples were shared between adjacent windows, so that nearly identical windows and windows from the same participant or trial could be present in both subsets. This compromises test-set independence, permits information from observations eventually assigned to testing to influence standardisation, can lead to optimistic estimates of accuracy and may have differential effects on architectures. The reported values and ordering of the model should therefore be interpreted as exploratory subject-dependent window-level results only. They do not estimate generalisation to unseen participants, cross-subject robustness or readiness for deployment.

A key additional validation that would be needed for future work would be a participant-resolved reanalysis with leave-one-subject-out or grouped participant-level cross validation. Participant groups should be pre-defined before pre-processing and window segmentation; normalisation parameters, hyperparameters or model selection, and GAN training should be fitted to training participants only; and the held-out test fold should consist of real windows from unseen participants only. Should be also examined the segmentations with lower overlap and non-overlap. Future work should involve larger and more diverse cohorts, less constrained acquisition, multi-rate fusion, and approaches that retain higher-frequency EMG information.

## Conclusion

The main findings and contributions of this study can be summarised as:

1. Public release of SDALLE: We have developed and publicly released a synchronised multimodal EMG–IMU locomotion dataset. We have documented the acquisition, preprocessing, and organization protocols to support reproducible wearable HAR research.
2. Subject-dependent cross-dataset comparison: The harmonised framework was used to describe established deep learning architectures across SDALLE and ENABL3S under consistent model-training choices. The partitioning was performed at the level of the overlapping window, and the comparison is protocol specific and does not reflect participant independent validation.
3. Protocol Specific Model Ordering: The attention-based CNN-LSTM variants on the subject dependent window level evaluation performed yielded the best metrics on both datasets. Such descriptive recurrence does not mean portable architectonic consistency and can be subjected to correlated windows and participant-specific information.
4. Measured performance of hybrid attention-enhanced models: Under the current randomised window-level split, attention-enhanced CNN-LSTM models registered the highest metrics and balanced precision-recall values. These results do not show superiority with unseen participants.
5. Observations on accuracy-complexity for the Hybrid protocol and attention-based architectures took longer to train, inference was relatively fast on the workstation used for the study. Such measurements are not a proof of real time operation on wearable hardware.
6. Exploratory role of GAN-based augmentation: We show that GAN augmentation has an impact on class proportions and generates small descriptive changes in the aggregate testing metrics under the implemented subject-dependent protocol. As no bias analysis by class, uncertainty assessment from repeated runs, or inferential statistical testing was performed, these results do not show reduced bias, better prediction stability, increased robustness, or better generalisation.
7. Scope and limitations: The study is limited by a small, demographically homogeneous cohort of healthy male participants, controlled laboratory acquisition, EMG downsampling, and a non-independent randomised window-level evaluation following full-dataset standardisation. Thus, the reported performance may be optimistic and needs to be further validated on a larger set of participants.

This study provides the SDALLE multimodal EMG–IMU dataset and a transparent exploratory comparison of well-established architectures under a subject-dependent window-level protocol. The present results do not support claims of cross-subject robustness or deployment readiness. Generalisation to unseen participants requires participant-level grouped validation with preprocessing and augmentation on the training fold only.

## Supplementary Information

Below is the link to the electronic supplementary material.

Supplementary Material 1

## Data Availability

The SDALLE EMG–IMU dataset generated during this study is publicly available via Zenodo at https://zenodo.org/records/14841611, including the raw sensor data and associated activity labels.
