# Supplementary material for: A multimodal EMG–IMU dataset and multi-dataset benchmark for deep learning-based human activity recognition
Source: Sci Rep. 2026 Aug 20;16:26180. doi: 10.1038/s41598-026-64847-4 (PMC13493917; doi:10.1038/s41598-026-64847-4)
Supplement: Supplementary file 1 [file 41598_2026_64847_MOESM1_ESM.pdf]

Faculty of Pharmacy
Egyptian-Russian University
Cairo, Egypt

The response of the scientific research ethical committee
Reference No. ERUFE-MR-26-001

May 18, 2026

Dear Dr. Mohamed Fawzy El-Khatib,

I am writing in response to your resubmission of your revised application for ethical approval of the study: **"A Multimodal EMG–IMU Dataset and Cross-Dataset Benchmark for Deep Learning Based Human Activity Recognition".**

The decision of the 13/5/2026 meeting was "your study has been **approved** on behalf of the Research Ethics Committee".

You should let us know if there are any significant changes to the proposal which raise any further ethical issues.

Please let us have a brief final report to confirm the research has been completed, and a copy of the published manuscript(s) of the study has been sent to the Scientific Research Committee.

Yours sincerely,

Professor Dr. Mahasen A. Radwan
Chair of the Committee
Faculty of Pharmacy

CamScanner
